# Alternative Splicing of a Receptor Intracellular Domain Yields Different Ectodomain Conformations, Enabling Isoform-Selective Functional Ligands

**DOI:** 10.1016/j.isci.2020.101447

**Published:** 2020-08-10

**Authors:** Fouad Brahimi, Alba Galan, Sean Jmaeff, Pablo F. Barcelona, Nicolas De Jay, Kurt Dejgaard, Jason C. Young, Claudia L. Kleinman, David Y. Thomas, H. Uri Saragovi

**Affiliations:** 1 Lady Davis Institute-Jewish General Hospital, McGill University, 3755 Côte St. Catherine, E-535, Montreal, QC H3T 1E2, Canada; 2 Department of Human Genetics, McGill University, Montreal, QC, Canada; 3 Department of Biochemistry, McGill University, Montreal, QC, Canada; 4 Department of Pharmacology, McGill University, Montreal, QC, Canada; 5 Department of Ophthalmology and Visual Science, McGill University, Montreal, QC, Canada

**Keywords:** Biological Sciences, Biochemistry, Structural Biology

## Abstract

Events at a receptor ectodomain affect the intracellular domain conformation, activating signal transduction (out-to-in conformational effects). We investigated the reverse direction (in-to-out) where the intracellular domain may impact on ectodomain conformation. The primary sequences of naturally occurring TrkC receptor isoforms (TrkC-FL and TrkC.T1) only differ at the intracellular domain. However, owing to their differential association with Protein Disulfide Isomerase the isoforms have different disulfide bonding and conformations at the ectodomain. Conformations were exploited to develop artificial ligands, mAbs, and small molecules, with isoform-specific binding and biased activation. Consistent, the physiological ligands NT-3 and PTP-sigma bind both isoforms, but NT-3 activates all signaling pathways, whereas PTP-sigma activates biased signals. Our data support an “in-to-out” model controlling receptor ectodomain conformation, a strategy that enables heterogeneity in receptors, ligands, and bioactivity. These concepts may be extended to the many wild-type or oncogenic receptors with known isoforms.

## Highlights

• In transmembrane proteins the intracellular domain impacts the ectodomain conformation
• Ectodomains with identical primary sequences can have different conformations
• Native ligands can bind conformations non-selectively but activate in a biased manner
• Synthetic ligands can bind and activate each receptor isoform selectively

## Graphical Abstract

**Figure:**
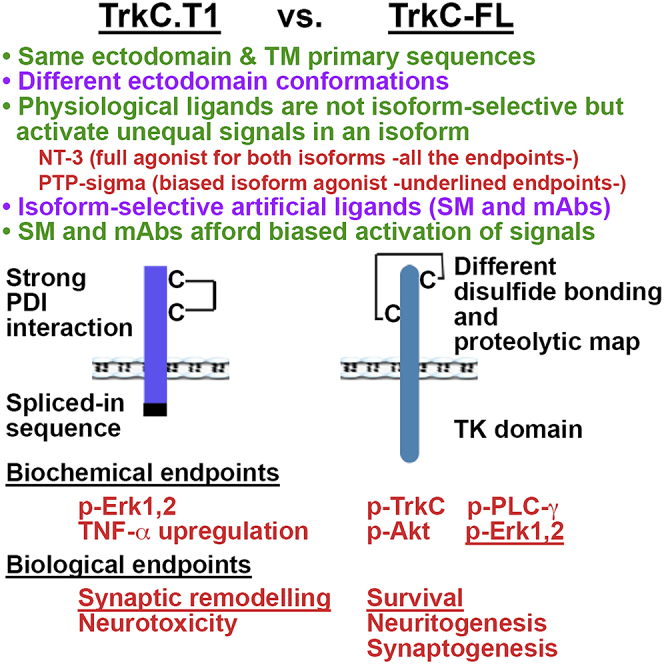

## Introduction

**Figure 1:**
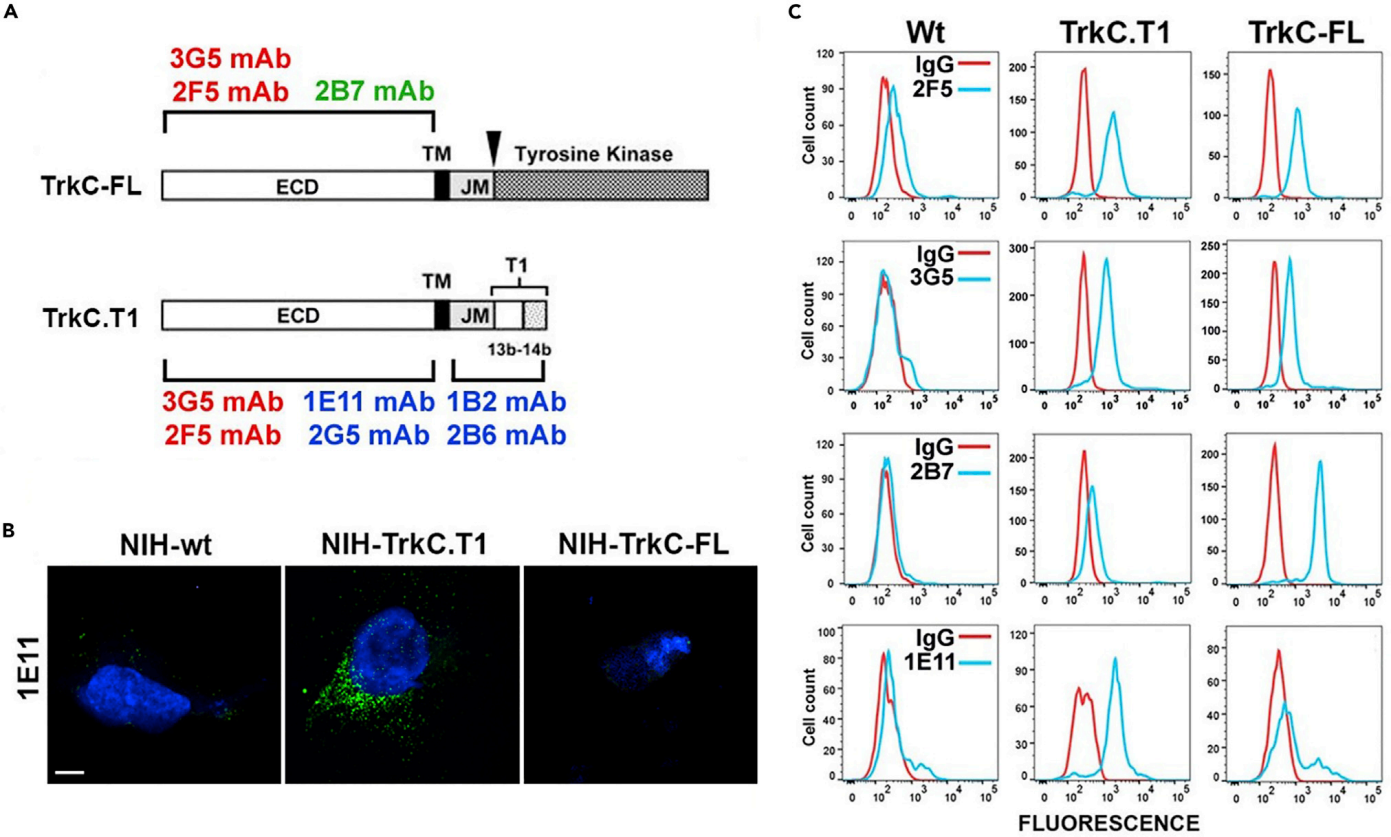
Binding of Isoform-Specific mAbs (A) Schematic representation of the full-length TrkC (TrkC-FL) and truncated TrkC.T1 receptors. ECD, extracellular domain; TM, transmembrane domain; JM, intracellular juxtamembrane domain. MAbs binding to the ECD of both isoforms (red), the ECD of TrkC-FL only (green) or the ECD of TrkC.T1 only (blue), or the ICD of TrkC.T1 only (blue) are shown. (B) Immunohistochemistry studies of wild-type or stably transfected NIH cells. Fluorescent microscopy shows that the mAb **1E11** bind to TrkC.T1 (green), and not to TrkC-FL expressing cells or to wild-type cells not expressing any TrkC isoform. Blue is nuclear staining using DAPI. Assays were repeated at least in three independent experiments, each in duplicate. Scale bar, 7 μm. (C) Cell surface receptors were studied by flow cytometry of live wild-type or stably transfected HEK293cells, without permeabilization. MAbs **2F5** and **3G5** bind to both TrkC-FL and TrkC.T1, mAb **2B7** binds only to TrkC-FL, and mAb **1E11** binds only to TrkC.T1. Mouse IgG primary (red lines) is the background negative control. Assays were repeated at least in four independent experiments.

The TrkC receptor is a key player in the development, selection, maintenance, health, phenotype, and function of motor neurons, vascular endothelium, and other cell types (Saragovi et al., 2009; Segal, 2003; Sendtner et al., 1996; Youn et al., 2003). The *trkC* gene can generate, through alternative splicing, mRNAs encoding for several isoforms (Tsoulfas et al., 1993). The full-length TrkC (TrkC-FL) protein intracellular domain has a tyrosine kinase catalytic domain, whereas the truncated TrkC.T1 protein lacks the kinase and most of the intracellular domain but gains a new exonic sequence (Figure 1A). The *trkC* mRNA splicing event changes the primary sequence of the intracellular domain, but the juxtamembrane, the transmembrane, and the ectodomain primary sequences remain identical (Esteban et al., 2006). Both TrkC-FL and TrkC.T1 are naturally occurring, but their expression patterns differ in health and in disease, and more importantly the isoforms transduce opposite signals.

TrkC-FL is expressed throughout life, and the tyrosine kinase signals are key to maintaining motor neuron health, phenotype, and function (Deinhardt and Chao, 2014). TrkC.T1 is expressed embryonically and plays a role in development (Palko et al., 1999; Tessarollo et al., 1997). TrkC.T1 remains expressed in some healthy adult tissues, although generally at lower levels than TrkC-FL (Bai et al., 2010; Esteban et al., 2006). In healthy adult spinal cord and retina TrkC.T1 expression is very low, but in neurodegenerative diseases that affect these tissues the expression is significantly upregulated, at early stages of pathology (e.g., ALS, glaucoma, retinitis pigmentosa). TrkC.T1 plays a deleterious role by stimulating overproduction of TNF-α to neurotoxic levels, thus contributing to disease onset and progression (Bai et al., 2010; Brahimi et al., 2016; Dorsey et al., 2006; Galan et al., 2017; Yanpallewar et al., 2012).

The primary amino acid sequence of TrkC-FL and TrkC.T1 ectodomains are identical, and both isoforms are bound by the same physiological ligands: the soluble growth factor NT-3 and the ectodomain of PTP-sigma expressed in an adjoining cell and binding in a heterotypic fashion. These ligands bind at non-overlapping receptor sites (D4-D5 and D1-D2 domains, respectively) and do not compete with each other (Coles et al., 2014; Takahashi et al., 2011). Similar high-affinity binding of NT-3 and PTP-sigma to the TrkC isoforms suggests highly conserved binding pockets.

However, there are differences in how ligands can activate the isoforms. NT-3 activates the TrkC-FL kinase with sustained kinetics, to signal via pErk and pAkt, to promote cell survival and differentiation and activates TrkC.T1 via Arf6/Rac1 (Esteban et al., 2006) and pErk (Bai et al., 2010) to promote TNF-α production to neurotoxic levels (Brahimi et al., 2016; Galan et al., 2017). In contrast, PTP-sigma binding causes synaptic reorganization equally well through either isoform, and this function is independent of an intracellular domain or any known direct signal transduction (i.e., TrkC without any intracellular domain also promotes synaptic reorganization) (Coles et al., 2014; Takahashi et al., 2011). PTP-sigma has no other ascribed function via TrkC, and no cell survival or neuronal differentiation has been reported.

Thus, we hypothesized that NT-3 and PTP-sigma may activate other signals via each TrkC isoform and predicted that biased signals may be identified, binding TrkC with isoform target selectivity. Ligands with isoform selectivity would be desirable in neurodegenerative diseases to selectively activate TrkC-FL neuroprotection and to selectively inhibit TrkC.T1 neurotoxicity (Saragovi et al., 2019). However, designing or screening agents with isoform selectivity is challenging given the conserved primary sequences and conservation of the binding pockets of the natural ligands.

Here, we report the development of monoclonal antibodies (mAbs) and small molecules as artificial ligands that are isoform selective and act as biased agonists to activate in full certain signals. Using these chemical tools, we show that TrkC-FL and TrkC.T1 ectodomains have different conformations. We also demonstrate a mechanism that explains the different conformations or folding in the isoforms. TrkC.T1 associates with protein disulfide isomerase (PDI), an enzyme that catalyzes different ectodomain disulfide bonding in TrkC.T1 compared with TrkC-FL.

The two different ectodomain conformations of TrkC-FL and TrkC.T1 are present *in vivo*, prompting the concept that the natural ligands NT-3 and PTP-sigma may functionally distinguish between these receptor conformations. NT-3 was confirmed as a full agonist for TrkC-FL (sustained kinetics, leading to survival and differentiation), whereas PTP-sigma acting via TrkC-FL is a biased agonist that transiently activates pErk1,2-mediated survival, without activating pAkt or cell differentiation. Moreover, NT-3 is an agonist of TrkC.T1-mediated promotion of TNF-α, whereas PTP-sigma does not activate this signal. This validates the concept that natural ligands binding either isoform can be functionally biased, a finding that is consistent with and validates physiologically the finding of artificial ligands (mAbs and small molecules) with biased function.

Together, the data support the concept that changes in the intracellular domain could affect the ectodomain conformation (“in-to-out” effects). This concept expands, in symmetric analogy, the established concept that ligand binding, or mutations, or protein-protein interactions at the ectodomain can affect the intracellular domain (“out-to-in” effects) (Changeux and Christopoulos, 2016; Dawson et al., 2005; Edelstein and Changeux, 2016; Lemmon et al., 2014). This in-to-out model may be expanded to other naturally occurring receptor isoforms or oncogenic receptors and may be used to identify isoform-selective naturally occurring or artificial biased functional ligands.

## Results

### Ectodomain-Binding TrkC-FL-Specific or TrkC.T1-Specific mAbs

We reported a mAb **2B7** that binds selectively to the TrkC-FL ectodomain but binds poorly to the TrkC.T1 ectodomain (Brahimi et al., 2016). To study the molecular basis for binding selectivity, we generated additional mAbs that are selective for each isoform or for all isoforms (data are summarized in Figure 1A).

Immunohistochemistry (Figure 1B) and flow cytometry (Figure 1C) studies show that mAb 1E11 is preferentially selective for the TrkC.T1 ectodomain. The same results were obtained using the 2G5 mAb (data not shown). MAbs 2F5 and 3G5 are pan-TrkC mAbs and bind to both TrkC.T1 and TrkC-FL ectodomains and control for receptor expression (Figure 1C).

The mAb 2B7 epitope is at the linker region between D5 domain and the transmembrane domain, adjacent to D5 (Guillemard et al., 2010), the domain where NT-3 binds (Urfer et al., 1998). NT-3 (Kd 50 pM) blocks the binding of mAb 2B7 (Kd 2 nM) to TrkC-FL, but mAb 2B7 does not block the binding of NT-3 (Guillemard et al., 2010) (Szobota et al., 2019). NT-3 does not block binding of mAbs 1E11 and 2G5 to the TrkC.T1 ectodomain and does not block the binding of mAbs 2F5 and 3G5 to TrkC.T1 or TrkC-FL ectodomains.

These data indicate that it is possible to generate several mAbs that are isoform selective either for TrkC-FL or for TrkC.T1 ectodomains, even though the isoforms have the same primary sequence. In contrast, NT-3 binds to both receptor isoforms.

Other mAbs specific to the TrkC.T1 neoepitope intracellular domain (mAbs 1B2 and 2B6) (Figure S1) were made and used as further controls for TrkC.T1 expression. To evaluate the specific binding of the mAbs *in vivo*, spinal cord tissues where both TrkC isoforms are expressed were studied. Neurons express TrkC-FL but do not express TrkC.T1. Activated glia express TrkC.T1 mRNA at the onset of neurodegenerative diseases such as amyotrophic lateral sclerosis (ALS) (Brahimi et al., 2016), glaucoma (Bai et al., 2010), and retinitis pigmentosa (Galan et al., 2017). The TrkC.T1-specific mAbs were used to study protein expression in wild-type versus the SOD1 G93A mouse model of ALS in spinal cords *in vivo*.

In healthy mice there was low expression of TrkC.T1, but the protein was significantly increased in SOD1 G93A mice at the onset of ALS, co-localizing with GFAP^+^-activated glia and astrocytes. TrkC.T1 protein expression data are consistent with reports that TrkC.T1 mRNA expression in glia is associated with disease progression (Bai et al., 2010; Brahimi et al., 2016; Dorsey et al., 2006; Galan et al., 2017; Yanpallewar et al., 2012). These data demonstrate that the conformationally different ectodomains are present in tissue *in vivo* and further confirm the selectivity of mAb **1E11** (TrkC.T1 ectodomain binding) and mAb 1B2 (binding to the spliced-in neoepitope of the TrkC.T1 intracellular domain) (Figure S1).

### Cysteine Modifications as the Basis for Ectodomain Conformational Differences and mAb Binding Specificity

The binding specificity of mAbs binding to TrkC ectodomains bearing identical primary sequence suggests differences in the ectodomain conformation. The presence of the full-length intracellular domain in TrkC-FL, the absence of most intracellular sequences in TrkC.T1, or the gain of the intracellular neoepitope in TrkC.T1 may be responsible.

We hypothesized that one possible conformational difference in the ectodomain may be at the disulfide bonds. Disulfide bonding is ultimately regulated by the entropy of the protein, and the distance of Cys residues, but alternate disulfide bonds can also form as intermediates during the folding process. In addition, endoplasmic reticulum (ER) enzymes such as PDI can break and reorganize disulfide bonding, until the final thermodynamically driven disulfide bonding configuration is achieved (Braakman and Hebert, 2013; Wallis and Freedman, 2013). ER transit time influences the ability of PDI to carry out disulfide reorganization of client proteins (Matsusaki et al., 2020).

We evaluated ER retention/export sequences (Geva and Schuldiner, 2014; Lee et al., 2004; Michelsen et al., 2005) in the intracellular domains of TrkC-FL and TrkC.T1 to potentially account for different ER transit times leading to different PDI interactions. The intracellular domain of TrkC-FL has seven ER export motifs and two ER retention motifs. The intracellular domain of TrkC.T1 has four ER retention motifs. An engineered construct deleting the whole intracellular domain (TrkC-Δ-ICD) has no intracellular ER retention/export motifs and serves as control (Figure S2).

From sequence analyses the prediction is that both TrkC-FL and TrkC-Δ-ICD transit rapidly through the ER and would have weak PDI associations, whereas TrkC.T1 transits more slowly through the ER and would have stronger PDI associations. This would result in TrkC-FL and TrkC-Δ-ICD adopting an intermediate disulfide bond pattern determined by kinetics, whereas TrkC.T1 would adopt a native thermodynamically driven PDI-mediated disulfide bond pattern and a different conformation (Kozlov et al., 2010b).

**Figure 2:**
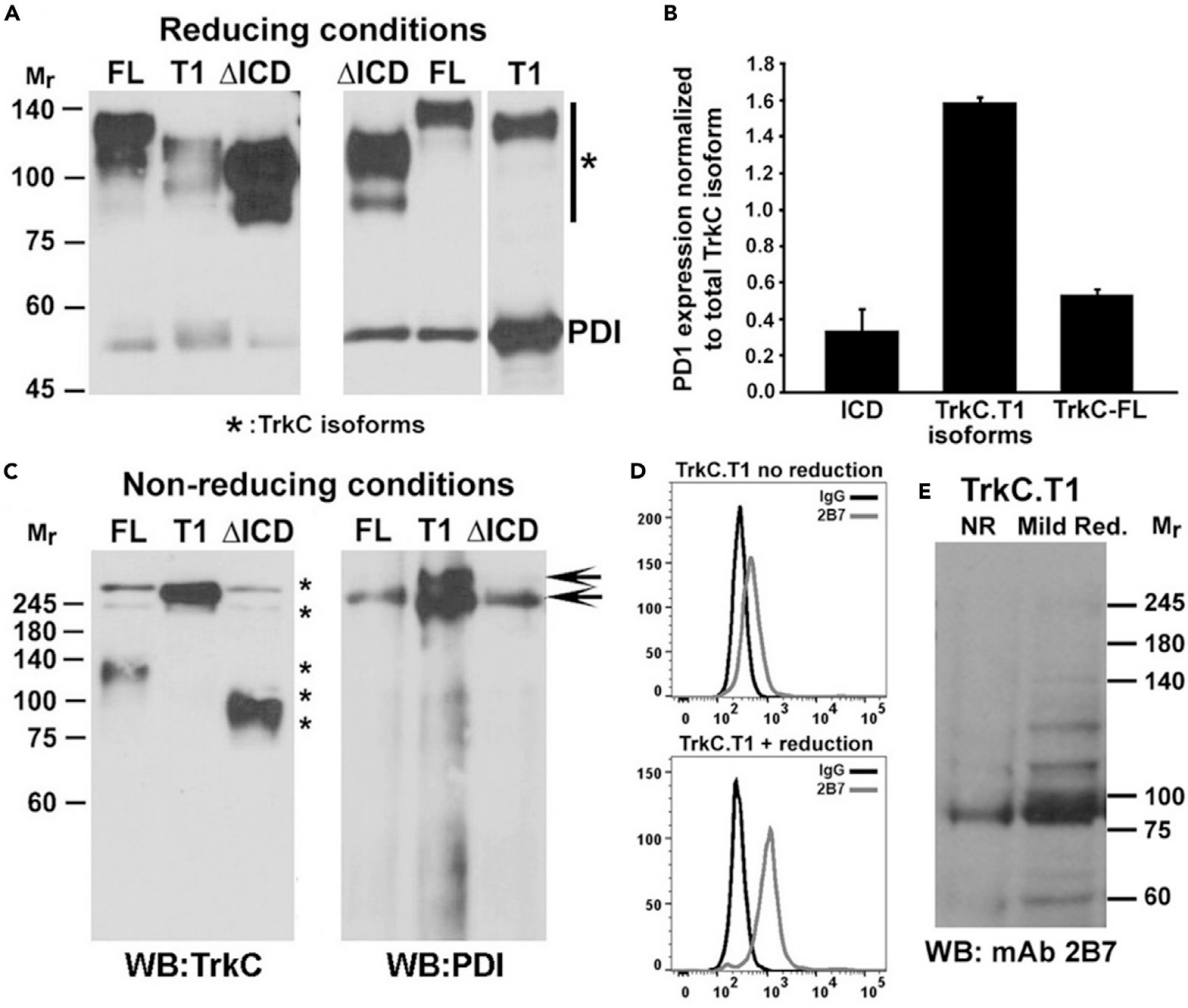
TrkC Isoforms Have Different Disulfide Bonding and Association with PDI All assays were repeated at least in three independent experiments. (A) Co-immunoprecipitation of PDI with TrkCT1 or TrkC-FL or TrkC-Δ-ICD HEK293 lysates. Immunoprecipitations using a pan-TrkC antibody were resolved by SDS-PAGE under reducing conditions, and membranes were immunoblotted with anti-pan-TrkC (C44H5) or with anti-PDI mAbs (results were replicated using two different anti-PDI mAbs). The different Mr of each TrkC isoform is due to the length of the ICDs. Heterogeneity is due to glycosylation. Left panel, same cell lysate equivalents loaded. Right panel, same TrkC levels loaded. (B) Quantification of PDI/TrkC ratios. TrkC.T1 associates with PDI at a high ratio or stoichiometry, approximately four times higher than the other isoforms. Data are mean ± standard deviation. (C) TrkC immunoprecipitates were resolved in non-reduced SDS-PAGE and immunoblotted with a pan-TrkC antibody or anti-PDI mAbs. Almost all the TrkC.T1 is in a high-molecular-weight complex co-migrating with PDI. (D) Flow cytometry show that TrkC-FL-specific 2B7 mAb does not bind to cell surface TrkC.T1 (top), but cell surface TrkC.T1 binding is gained (bottom) after exposing live cells to mild reduction. (E) Western blots of whole-cell lysate samples show that TrkC-FL-specific 2B7 mAb does not bind to non-reduced TrkC.T1 (NR), but binding is gained after mild reduction of cell lysates.

To evaluate this prediction, co-immunoprecipitations quantified the association of each TrkC isoform with PDI. TrkCT1, TrkC-FL, or TrkC-Δ-ICD was immunoprecipitated with anti-pan-TrkC (recognizing all isoforms), and samples were studied by western blotting using two different anti-PDI mAbs or two different anti-pan-TrkC mAbs (Figures 2A–2D).

PDI and TrkC.T1 are associated at a high ratio or at high stoichiometry, whereas PDI is associated with TrkC-FL or with TrkC-Δ-ICD at a significantly lower level (Figure 2A). When standardized to the total TrkC loaded in each lane, TrkC.T1 has a 4-to 6-fold higher associated PDI than the other two isoforms (Figure 2B).

Analyses of the samples under non-reducing conditions demonstrate that the majority of the PDI⋅TrkC.T1 is detected as a complex, at a mass above 245 kDa (Figure 2C left panel). This is consistent with the reaction mechanism of PDI (Kozlov et al., 2010a) in which it forms transient disulfide bonds with substrates (Braakman and Hebert, 2013). A minority of the associated PDI⋅TrkC.T1 is non-covalent or of low stoichiometry, and a ladder of free PDI protein is also detected (Figure 2C right panel).

In contrast, PDI⋅TrkC-FL and PDI⋅TrkC-Δ-ICD association is mostly non-covalent; these TrkC isoforms are detected in their free state under non-reducing conditions. Very low levels of PDI⋅TrkC-FL and PDI⋅TrkC-Δ-ICD are detected as a stable complex, at a mass above 245 kDa (Figure 2C left panel). The Mr heterogeneity of the TrkC isoforms is expected from their different intracellular domain mass (Figures 2A and 2C).

Consistent with our predictions that TrkC-FL and the TrkC-Δ-ICD isoforms share a related conformation, mAb **2B7** binds to both TrkC-FL and TrkC-Δ-ICD but less efficiently to TrkC.T1. These data suggest and that TrkC.T1 conformation is different from that of TrkC-FL and TrkC-Δ-ICD.

To evaluate this concept, we tested whether mAb **2B7** binding is sensitive to mild reducing agents. In flow cytometry assays using live cells, mild reduction of TrkC.T1 results in gain of mAb **2B7** binding at the cell surface (Figure 2D). This shows that breaking at least some of the disulfide bonds in TrkC.T1 unmasks the 2B7 epitope and supports the view that TrkC.T1 has a different disulfide configuration. Western blot analyses support a similar conclusion, as mild reduction of TrkC.T1 (without full denaturation of the sample) results in increased mAb 2B7 binding to TrkC.T1 (Figure 2E). The flow cytometry and western blot assays are consistent in that mild reduction significantly enhances mAb **2B7** binding to TrkC.T1.

### Conformational Differences in the Ectodomains of the Isoforms

Immunoprecipitations of TrkC isoforms (using a pan-TrkC mAb) were subjected to trypsin peptide mapping and studied by liquid chromatography-mass spectrometry (LC/MS). The same samples were studied under reducing and non-reducing conditions, to detect tryptic fragments that remain paired under non-reducing conditions. The datasets were analyzed using bioinformatic tools (Scaffold 4.8.9).

**Figure 3:**
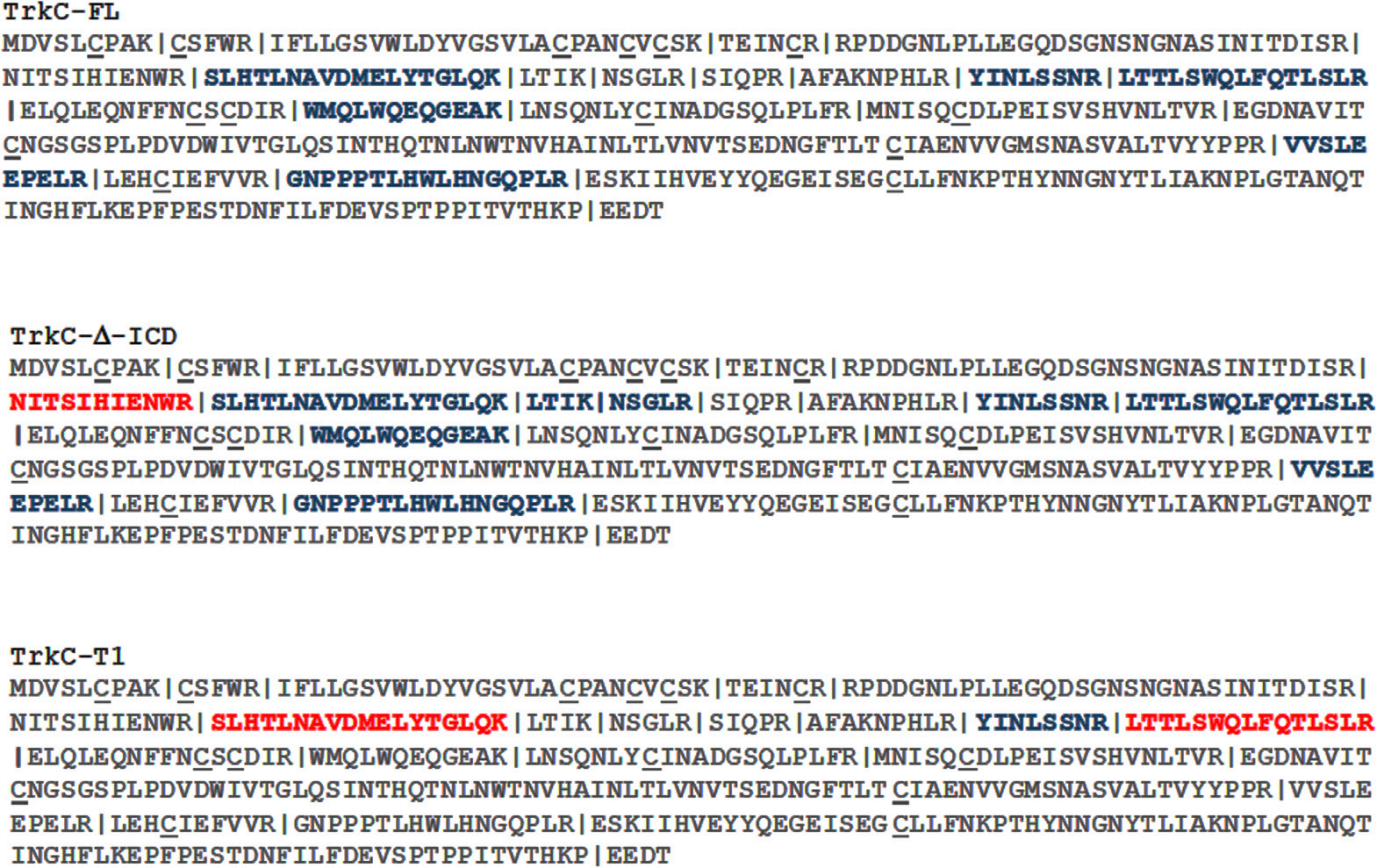
TrkC Isoforms Have Different Disulfide-Bonding Patterns HEK293 lysates expressing TrkC isoforms were immunoprecipitated using a pan-TrkC mAb and the proteins were digested with trypsin. Samples were studied by LC/MS, with half of each sample first subjected to reduction/alkylation of S-S bonds (half of each sample was not). **Red letters**: peptides detected under reducing conditions only. **Blue letters**: peptides detected under both reducing and non-reducing conditions. Dashed vertical lines: trypsin cut sites. Cysteines are underlined in gray. The fragmentation patterns of TrkC-FL and TrkC-Δ-ICD are highly related and very different from the fragmentation pattern of TrkC.T1.

The peptide maps for the TrkC-FL and TrkC-Δ-ICD isoforms are similar, suggesting a similar conformation. TrkC-FL and TrkC-Δ-ICD isoforms displayed a variety of intra-chain disulfide bonds. However, their map differs significantly from the map of the TrkC.T1 isoform (Figure 3). Notably, after digestion TrkC.T1 is the only isoform where homodimer fragments are detected (LTTLSWQLFQTLSLR fragment), under non-reducing conditions, although they contain no cysteines. This suggests that in TrkC.T1 the fragments remain aggregated. These data indicate that the conformation of the TrkC-FL and TrkC-Δ-ICD isoforms is different from that of TrkC.T1 and that the disulfide bonds are different.

Overall, the data in Figures 2 and 3 confirm that each TrkC isoform has a distinct conformation, disulfide bonding, and PDI association and indicate that splicing of the intracellular domain can cause conformational differences at the ectodomain. The data in Figure 1 support this conclusion. Although the receptor isoforms have an identical primary amino acid sequence, and the natural ligand NT-3 does not discriminate between isoforms, structural differences may be exploited to generate isoform-selective ligands.

### Ectodomain-Binding Isoform-Specific mAbs Can Be Agonistic

Next, we tested the isoform-specific mAb ligands for activation of signal transduction in assays quantifying cell survival and differentiation (TrkC-FL function) and stimulation of TNF-α production (TrkC.T1 function) (Brahimi et al., 2016). NT-3 is a non-discriminating agonist of both isoforms and serves as positive control.

**Figure 4:**
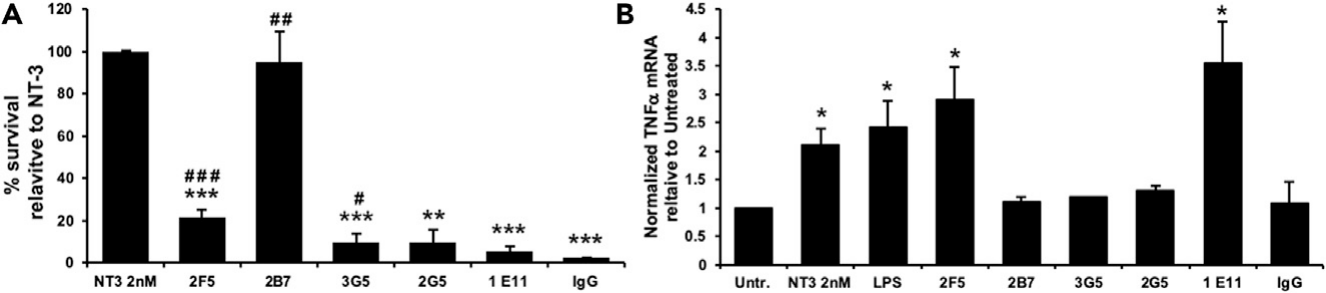
Isoform-Selective mAb Ligands Have Isoform-Selective Bioactivity All assays were repeated at least in three independent experiments, each in triplicate. Dose-responses not shown, for simplicity (for further information see Figure S5). Data are mean ± standard deviation. One symbol p < 0.05, two symbols p < 0.01, three symbols p < 0.001. (A) HEK293 cells expressing TrkC-FL receptors were cultured in serum-free media (to induce death) and were supplemented with the indicated ligands (40 nM for mAbs, 2 nM for NT-3). Cell survival was determined after 72 h by MTT colorimetric assays (similar results were obtained after 48 h, not shown). Untreated (vehicle) and mouse IgG are controls (0% survival), NT-3 at the optimal 2 nM dose is set to 100% survival. The symbol # shows significance versus mouse IgG control. The symbol ∗ shows significance versus NT-3 positive control. MAb **2B7** significantly promotes cell survival, to levels similar to NT-3. MAbs **2F5** and **3G5** also promote cell survival but to a lower degree than NT-3. MAbs **2G5**, and **1E11** do not promote cell survival and are not different from mouse IgG control (one-way ANOVA with Tukey-Kramer multiple comparisons). (B) TNF-α mRNA quantified by quantitative real-time PCR after 6 h of treatment in glial rMC-1-TrkC.T1. The values were standardized to the vehicle control (untreated, set to 1). NT-3 (2 nM) and LPS (1 μg/mL) are positive controls. MAbs (at 40 nM) **2F5** and **1E11** significantly increase TNF-α mRNA levels (∗p < 0.05 versus untreated or mouse IgG controls). The mAbs **2B7**, **3G5**, and **2G5** do not increase TNF-α and are not different from untreated or mouse IgG controls (two-tailed t test).

**NT-3** activates cell survival and cell differentiation. MAb **2B7** activates TrkC-FL to promote cell survival with maximal efficacy similar to NT-3 (Figure 4A) but does not promote cellular differentiation (Guillemard et al., 2010). Therefore, mAb 2B7 is a biased agonist for TrkC-FL isoforms. In addition, mAb **2B7** does not activate TrkC.T1 and does not induce TNF-α (Figure 4B) (as expected because it does not bind cell surface TrkC.T1). In sum, mAb 2B7 is a TrkC-FL-specific agonist biased to cell survival and it is more receptor specific than NT-3 because it does not bind to TrkC.T1 (Brahimi et al., 2016) or p75NTR (Guillemard et al., 2010).

MAb **2F5** binds to both TrkC-FL and to TrkC.T1 and functionally is an agonist for both isoforms. MAb **2F5** promotes limited cell survival with significantly lower efficacy than NT-3 (Figure 4A) but promotes TNF-α production to levels similar to NT-3 (Figure 4B). The effective molar concentration for mAb 2F5 is the same for each isoform, indicating equivalent potency. However, relative to NT-3, mAb 2F5 activates the TrkC.T1 isoform with higher efficacy. MAb 3G5 also binds to both TrkC-FL and TrkC.T1 and promotes limited cell survival via TrkC-FL, without promoting TNF-α production via TrkC.T1. Hence the mAbs can be partial agonists and they can activate the isoforms asymmetrically in spite of comparable binding to both isoforms.

MAb 1E11 is TrkC.T1 specific and activates TrkC.T1 with high efficacy, inducing TNF-α production to levels similar to control NT-3 or control LPS (Figure 4B). Silencing TrkC.T1 mRNA expression (using pLKO-1 viral vectors expressing shRNA (Brahimi et al., 2016) prevents TNF-α induction by mAb 1E11 and by NT-3 but does not prevent TNF-α induction by LPS control (Figure S3). These data demonstrate that TNF-α production is dependent on TrkC.T1 expression and is ligand dependent. In cellular controls, the TrkC.T1-specific mAb 1E11 does not promote cell survival through TrkC-FL (Figure 4A), as expected because it does not bind this isoform. Hence mAb 1E11 is a TrkC.T1-specific agonist.

### Ectodomain-Binding Isoform-Specific Small Molecules Can Be Agonistic

To expand the proof of concept of isoform-selective agonistic ligands, we studied small molecules (**3Aa** and **1Aa**) and their labeled analogs (3Aa-FITC and 1Aa-FITC, containing a fluorescein label). We previously reported in quantitative flow cytometry assays that **3Aa** and **1Aa** are ligands binding to the TrkC-FL ectodomain. Small molecule TrkC ligands had not been evaluated for isoform specificity, because at the time the concept of different isoform structures and differential ligand binding had not been rationalized.

**Figure 5:**
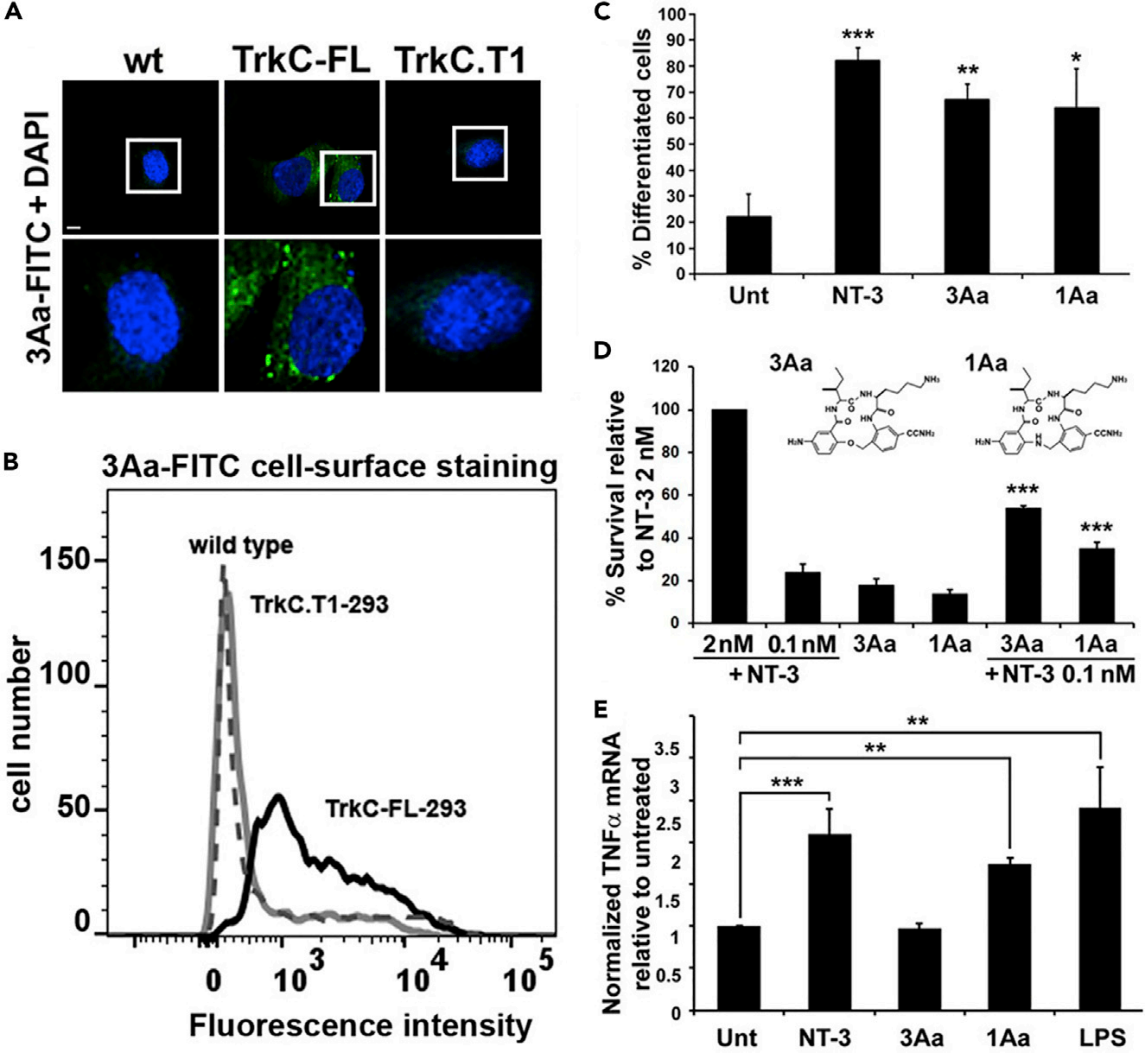
Binding and Bioactivity of Isoform-Specific Small Molecule Ligands All assays were repeated at least in three independent experiments, each in triplicate. Data are mean ± standard deviation. Symbols indicate significance ∗∗∗p < 0.001, ∗∗p < 0.01, ∗p < 0.05 (two-tailed t test). The binding and bioassays shown for **3Aa** and **1Aa** are at 10 μM (as per Zaccaro et al., 2005). Optimal NT-3 is 2 nM, and 0.1 nM NT-3 is suboptimal (for dose-dependent bioassay data see Figure S5). (A) Binding assays by immunohistochemistry in HEK293 cells. Merged DAPI and FITC channels are shown. The bottom panels show a higher magnification of the top panel areas within the white rectangles. Scale bar, 7 μm. Fluorescein-conjugated **3Aa** (3Aa-FITC) binds predominantly to TrkC-FL and binding is lower or undetectable to TrkC.T1. (B) Binding assays by quantitative flow cytometry. Ligand binding to the cell surface, using live, non-permeabilized HEK293 cells. 3Aa-FITC binds to TrkC-FL. (C) Biological assays of cell differentiation via TrkC-FL. **3Aa** and **1Aa** induce differentiation of nnr5-TrkC-FL cells, to levels similar as 2 nM NT-3 control. Differentiation was determined as percentage of cells with neurites (defined as length >2 cell bodies). Significance shown is versus untreated. (D) Biological assays of cell survival via TrkC-FL. **3Aa** and **1Aa** promote the survival of HEK293-TrkC-FL cells induced to die by culture in serum-free media. Data are standardized to 2 nM NT-3 100% survival, and untreated 0% survival. **3Aa** and **1Aa** can potentiate the suboptimal action of 0.1 nM NT-3 (∗∗∗p < 0.001 significance versus 0.1 nM NT-3 control). For simplicity, some statistics are not shown in the graph: (i) all agents are significant versus untreated, (ii) the survival promoted by **3Aa** and **1Aa** is not different from suboptimal 0.1 nM NT-3 but is significantly lower than optimal 2 nM NT-3. Structures of **3Aa** and **1Aa** are shown. (E) Biological assays of TNF-α induction via TrkC.T1. **1Aa** induces TNF-α mRNA, similar to controls NT-3 (2 nM) or LPS (1 μg/mL). In contrast, **3Aa** does not induce TNF-α mRNA, as expected since it does not bind efficiently to TrkC.T1 (see A and B).

Here, we demonstrate isoform specificity by small molecule TrkC ligands **3Aa** and **1Aa**, in terms of receptor binding (Figures 5A and 5B) and in terms of receptor activation (Figures 5C–5E).

In immunohistochemistry assays (Figure 5A) and in quantitative flow cytometry assays (Figure 5B) **3Aa-FITC** binds to cells expressing TrkC-FL but it does not bind to cells expressing TrkC.T1 above the background of control wild-type cells not expressing any TrkC isoforms. Previous work showed that **3Aa-FITC** does not bind to other Trk-receptor family members (Zaccaro et al., 2005); hence, **3Aa** binds with TrkC-FL isoform specificity, and such selectivity is remarkable for a small molecule. In contrast, **1Aa-FITC** binds to both TrkC isoforms (Table 1).

**Table 1:** Summary of TrkC Ligands Binding at the Ectodomain

Fold-Increase in Ligand Binding over Controls			
	Ligand	TrkC-FL	TrkC.T1
**Not Isoform Selective**			

1	NT-3	9	7
2	2F5 mAb	7.2	7.4
3	3G5 mAb	3	4.4
4	1Aa	6	5
5	PTP-sigma	5	3.7

**Selective TrkC-FL**			

6	2B7 mAb	16.3	–
7	3Aa	12	–

**Selective TrkC.T1**			

8	1E11 mAb	1.3	11.5
9	2G5 mAb	–	14

Quantitative flow cytometry assays of ligand binding at the cell surface of cells stably transfected to express TrkC-FL or TrkC.T1. The data are fold-increase in mean fluorescence intensity (average n ≥ 3) over all controls. Controls used are wild-type cells (untransfected), non-binding IgG, and labeled small molecule analogs that bind other targets. Data exemplified in Figures 1C and CS4 and previous work (Zaccaro et al., 2005). Binding is done at ligand-saturation concentrations, under conditions that yield low non-specific background staining, and high signal/noise ratios.

**NT-3** binds to both isoforms. **PTP-sigma** binds to both isoforms. **2F5** and **3G5** mAbs bind both isoforms. **1E11** and **2G5** mAbs bind selectively to TrkC.T1, and mAb **2B7** binds selectively to TrkC-FL. Small molecule **3Aa** binds TrkC-FL (this paper and Zaccaro et al., 2005) but does not bind to TrkC.T1. In contrast, small molecule **1Aa** binds to TrkC-FL (this paper and Zaccaro et al., 2005) and also to TrkC.T1. Not listed are mAbs **1B2** and **2B6** that bind selectively to the intracellular neoepitope of TrkC.T1.

NT-3-biotin was detected using avidin-fluorescein secondary. **1Aa** and **3Aa** were directly labeled by conjugation with fluorescein (1:1 fluorescein-per molecule). Mouse mAbs were detected using anti-mouse secondary. **PTP-sigma** was detected using anti-human secondary. Differences in the absolute fluorescence intensity can be ascribed to the different number of fluorescein conjugates and to different signal amplification and the stoichiometry of multiple secondary binding to a primary.

Biological assays evaluated cell differentiation and cell survival signals in TrkC-FL-expressing cells. Treatment with **3Aa** and **1Aa** induces significant cellular differentiation to levels comparable with NT-3 (Figure 5C), but with lower potency (10–50 μM for small molecules versus 0.2–2 nM for NT-3). **3Aa** and **1Aa** also promote low but significant survival compared with untreated control cultures (Figure 5D) as reported (Zaccaro et al., 2005). In addition, **3Aa** and **1Aa** potentiate the action of 0.1 nM NT-3 (∗∗∗p < 0.001 significance versus each ligand alone).

In sum, **3Aa** and **1Aa** are TrkC-FL partial agonist small molecules with biased signaling. They promote TrkC-FL function of cell survival with low efficacy and TrkC-FL function of cellular differentiation with good efficacy. However, their potency is low and activity requires relatively high concentrations.

The small molecules were then evaluated for TrkC.T1 signaling. Biological assays show that **3Aa** does not activate TrkC.T1, as expected, since **3Aa** does not bind to TrkC.T1. On the other hand, **1Aa** activates TrkC.T1 to induce TNF-α production to levels comparable with that of control NT-3 (Figure 5E). Therefore, functionally **3Aa** is a TrkC-FL-specific agonist, whereas **1Aa** is an agonist that is not isoform specific.

It is somewhat surprising that **3Aa** and **1Aa** have different isoform selectivity, because they have a closely related chemical structure with the only difference being that one has an amine and the other an ether in the ring (Figure 5D). Isoform selectivity and structure-activity relationship studies for >50 small molecule TrkC ligands (Brahimi et al., 2010; Chen et al., 2009; Zaccaro et al., 2005) will be reported elsewhere.

Isoform binding and functional selectivity achieved with artificial ligands (mAbs and small molecules) further supports the notion that there are structural differences in the receptors despite their invariant primary sequence. Structural differences suggest a refined control of receptor activation, which would seem necessary to maintain normal physiology, especially given that the natural ligand NT-3 can activate both isoforms.

### A Non-neurotrophin Endogenous Ligand Functionally Interacts with TrkC Isoforms in a Biased Manner

The ectodomain of PTP-sigma is reported to bind to TrkC-FL and TrkC.T1 and to promote synaptic reorganization. Synaptic reorganization induced by PTP-sigma was mediated also by TrkC-Δ-ICD, indicating that the effect is independent of any TrkC intracellular domain and of any reported TrkC-signal transduction (Coles et al., 2014; Takahashi et al., 2011).

To understand the PTP-sigma signaling mechanisms, we evaluated binding and activation for each TrkC isoform. In flow cytometry assays using live cells, the ectodomain of PTP-sigma ectodomain binds to all cell surface TrkC isoforms (Figure S4) as reported by others (Coles et al., 2014; Takahashi et al., 2011). Hence, PTP-sigma binding is impervious to the different conformation of each TrkC ectodomain. Moreover, PTP-sigma (Kd 9.3 ± 1.2 nM) and NT-3 do not cross-compete each other's binding (Coles et al., 2014; Takahashi et al., 2011), and this is consistent with PTP-sigma binding at the D1/D2 domains (Coles et al., 2014; Takahashi et al., 2011), whereas NT-3 binds at the D5 domain (Urfer et al., 1995, 1998).

**Figure 6:**
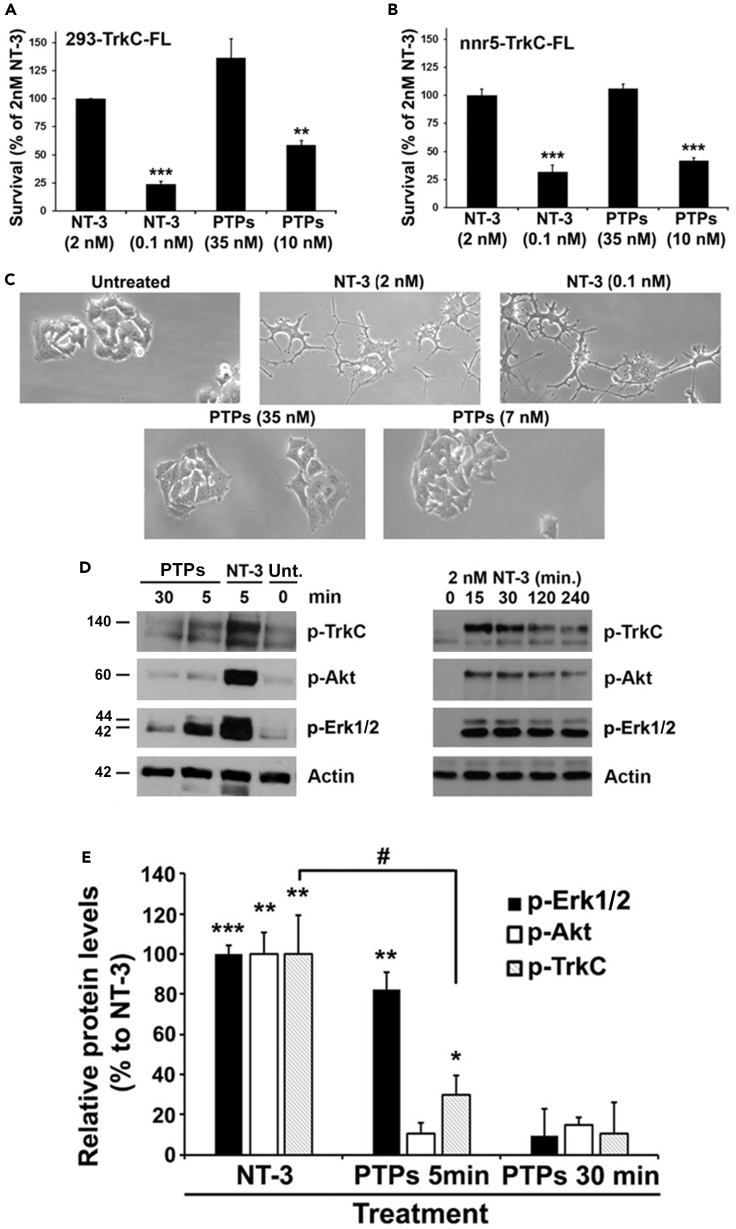
Biased Agonism by a Non-Neurotrophin Endogenous Ligand of TrkC-FL, the Ectodomain of PTP-Sigma PTP-sigma ectodomain binds to all TrkC isoforms (Figure S4) and was evaluated for bioactivity via TrkC-FL. Data are standardized to 2 nM NT-3 = 100% survival, untreated = 0% survival. (A) PTP-sigma promotes the survival of 293-TrkC-FL cells induced to die by culture in serum-free media. PTP-sigma (35 nM optimal dose) is as effective as NT-3 (2 nM optimal dose), but dose-dependent studies show that in this biological endpoint it is 20- to 50-fold less efficient than NT-3 (significance versus optimal NT-3. Data are mean ± standard deviation. Symbols indicate significance ∗∗∗p < 0.001, ∗∗p < 0.01; two-tailed t test). Similar data were obtained using NIH3T3-TrkC-FL cells. (B) PTP-sigma and NT-3 promote the survival of nnr5-TrkC-FL cells (a PC12 cell variant, expressing TrkC-FL and p75^NTR^). The effect of PTP-sigma and NT-3 is dose dependent (significance versus optimal NT-3, ∗∗∗p < 0.001; two-tailed t test). (C) In spite of promoting survival signals, PTP-sigma binding does not promote *any* cell differentiation or neuritic outgrowth in nnr5-TrkC-FL cells, whereas differentiation is clearly promoted by NT-3. (D) Biochemical signals in HEK293-TrkC-FL cells (n = 3 independent assays). The PTP-sigma signal is transient and decreases to baseline by 30 min, whereas the NT-3 signal is sustained for hours. p-Erk1,2 is significantly stimulated by PTP-sigma and is not statistically different from NT-3 at the 5-min time point. (E) Quantification showing significance versus baseline (∗∗∗p < 0.001, ∗∗p < 0.01, or ∗p < 0.05; one-way ANOVA with Tukey-Kramer multiple comparisons). The pTrkC signal is also activated transiently at the 5-min time point, to a significantly lower degree than NT-3 (#p < 0.05).

In biological assays using TrkC-FL-expressing cells stressed by culture in serum-free media, PTP-sigma promotes cell survival. In these assays, 35 nM PTP-sigma induces a maximal survival that is similar to optimal 2 nM NT-3 control. Although the efficacy of PTP-sigma is equal to NT-3, on a molar basis PTP-sigma is 20- to 50-fold less potent than NT-3 (Figures 6A and S5). The efficacy and potency of PTP-sigma at promoting cell survival via TrkC-FL is the same whether or not cells co-express the NT-3 co-receptor p75NTR (Figure 6B). However, PTP-sigma acting through TrkC-FL does not promote cellular differentiation whatsoever (Figure 6C).

The biological endpoints triggered by PTP-sigma binding to TrkC-FL are associated with signal transduction that is biased to pErk1,2 (Figure 6D, quantified in 6E). Compared with NT-3, PTP-sigma activates pErk1,2 signals to a maximal level that is not different from NT-3. However, the PTP-sigma signals are transient and last 5–7 min, whereas NT-3 signals are sustained for hours (Guillemard et al., 2010; Ivanisevic et al., 2003). Moreover, PTP-sigma also transiently activates pTrkC signals significantly over vehicle control, but to a significantly lower degree than NT-3.

The kinetics of the biochemical signals induced by PTP-sigma in TrkC-FL-expressing cells are consistent with promotion of survival without promotion of differentiation (Saragovi et al., 1998; Zhang et al., 2000). Ligand-induced receptor internalization may be relevant to these biological signals (Saragovi et al., 1998). The pErk-biased signal transduction of PTP-sigma is analogous to mAb 2B7 (Brahimi et al., 2016), a ligand which also induces cell survival but not differentiation (Guillemard et al., 2010). However, there are differences between PTP-sigma and mAb 2B7 in that the latter induces pErk-activation with sustained kinetics, more similar to NT-3 kinetics.

**Figure 7:**
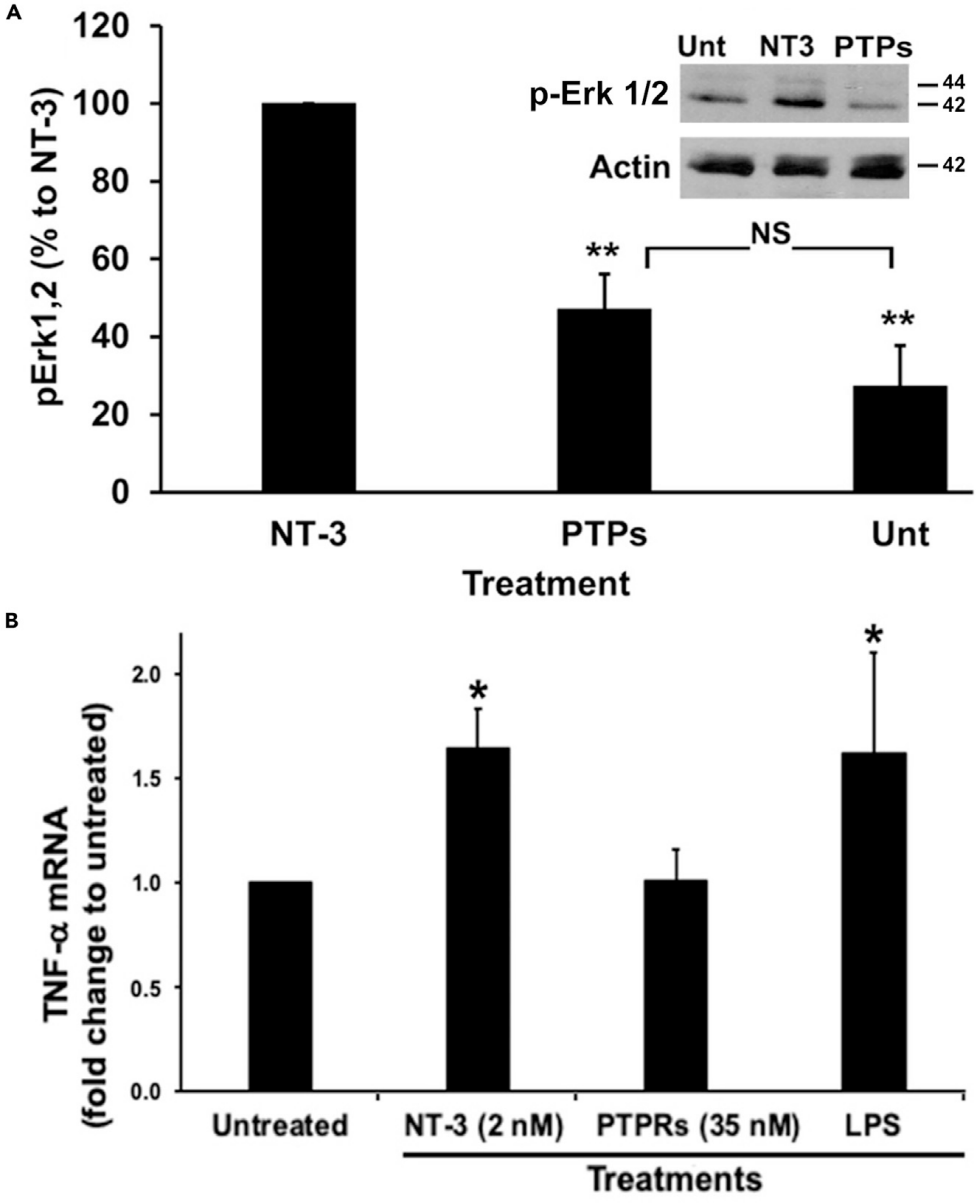
The Ectodomain of PTP-sigma Does Not Activate Key Functions of TrkC.T1 PTP-sigma ectodomain binds to all TrkC isoforms (Figure S4) and was evaluated for TrkC.T1-mediated bioactivity in rMC-TrkC.T1 cells (expressing TrkC.T1). Data are standardized to baseline untreated control or NT-3 positive control. Data are mean ± standard deviation. Symbols indicate significance, two-tailed t test. (A) PTP-sigma does not induce pErk1,2 above vehicle control, whereas positive control NT-3 does. An example of a blot is shown as inset. Quantification from three independent experiments, ∗∗p < 0.01 versus NT-3 control. NS, not significant. (B) PTP-sigma does not induce TNF-α mRNA above vehicle control. Positive control NT-3 induces TNF-α mRNA, and this is prevented by silencing TrkC.T1 expression (Figure S3). Another positive control, LPS (1 μg/mL), activates TNF-α mRNA production acting via TOLL-like receptors, and the LPS effect is not affected by silencing TrkC.T1 expression (Figure S3). ∗p < 0.05 versus untreated control.

In biological assays using TrkC.T1-expressing cells, PTP-sigma does not promote activation of pErk (Figure 7A) and does not promote TNF-α production (Figure 7B). In contrast, the positive control NT-3 activates pErk and promotes TNF-α production as an agonist of TrkC.T1.

These biochemical and biological endpoints are dependent on TrkC.T1 expression, because silencing TrkC.T1 prevents NT-3-induced pErk and TNF-α production (Figure S3). Consistent with this control, we had previously reported that silencing TrkC.T1 expression or inhibiting TrkC.T1 activation prevents TNF-α over-production and neurodegeneration *in vivo* (Galan et al., 2017).

Hence, PTP-sigma is not an agonist for the TrkC.T1-mediated endpoints of pErk activation (biochemical signals) and promotion of TNF-α production (biological signals), even though it binds to this isoform. The identification of a biased natural ligand PTP-sigma that functionally discriminates between TrkC isoforms provides a physiological counterpart to the isoform-selective TrkC artificial ligands (mAbs and small molecules).

In sum, NT-3 binds both isoforms and is a full agonist for both isoforms, whereas PTP-sigma binds both isoforms but functionally is a biased agonist in terms of receptor selectivity (only TrkC-FL activation) as well as in terms of biological endpoints (only survival is promoted). However, others have shown that PTP-sigma promotes synaptic reorganization equally well through either isoform, but this endpoint is independent of any TrkC-intracellular domain and has no known TrkC-intracellular signals (Coles et al., 2014; Takahashi et al., 2011).

Together, the data indicate that the isoform ectodomain conformations are present *in vivo* (Figure S1), that the isoforms are physiologically relevant, and that the endogenous ligands NT-3 and PTP-sigma do not activate equally and functionally discriminate between isoforms. These concepts are further supported by the discovery of artificial ligands whose binding and function also discriminate between isoforms.

A summary of binding data is shown in Table 1, and a summary of biological data is shown in Table 2. For all ligands, dose-response curves of bioactivity were evaluated, and the effective concentrations (EC) were determined. In the manuscript, for simplicity we mainly show the optimal ECs, which correspond to approximately 2 nM NT-3, 40 nM for mAbs and PTP-sigma, and 10 μM for small molecules. MAb **2B7** and PTP-sigma have a maximal efficacy that is comparable with that of NT-3, but they have lower potency (EC_50_ 2 nM for **2B7** and 8 nM for PTP-sigma, versus EC_50_ 0.2 nM for NT-3). MAb **2F5** has lower efficacy and potency. Small molecules **3Aa** and **1Aa** have significantly lower potency and low efficacy. However, **3Aa** and **1Aa** increase the efficacy of 0.1 nM NT-3, from 25% to 65% and 71%, respectively (Figure S5).

**Table 2:** Functional Efficacy of TrkC Ligands Binding at the Ectodomain

	Ligand	TrkC-FL Activity		TrkC.T1 Activity
		Survival	Different	TNF-α mRNA
**Not Isoform Selective**				

1	NT-3	**100**	**100**	**100**
2	2F5 mAb	**22**	N.S.	**172**
3	3G5 mAb	N.S.	N.S.	N.S.
4	1Aa	**∗71**	**78**	**64**

**Selective TrkC-FL**				

5	2B7 mAb	**95**	N.S.	N.S.
6	3Aa	**∗65**	**82**	N.S.
7	PTP-sigma	**105**	N.S.	N.S.

**Selective TrkC.T1**				

8	1E11 mAb	N.S.	N.S.	**236**
9	2G5 mAb	N.S.	N.S.	N.S.

Cell survival and differentiation are the endpoints for TrkC-FL agonism. The endpoints for TrkC.T1 agonism are pErk activation and promotion of TNF-α mRNA and protein. Efficacy and potency are relative to 2 nM NT-3 (set to 100%). All ligands exhibit a dose-dependent pharmacological effect (e.g., Figures 6A and S5). The *in vitro* effective concentrations (EC) for each ligand, depending on the assay, are at 0.5–2 nM for NT-3, 20–40 nM for mAbs and PTP-sigma, and 10–50 μM for small molecules.

**NT-3** agonizes all isoforms. **PTP-sigma** is a biased agonist of TrkC-FL and promotes cell survival but not cell differentiation. **PTP-sigma** binds to TrkC.T1 but does not activate the TrkC.T1 endpoints studied here. However, PTP-sigma activates the endpoint of synaptic reorganization via TrkC in a non-isoform selective manner and independent of known TrkC signals (Coles et al., 2014; Takahashi et al., 2011). MAb **2F5** activates both TrkC isoforms (TrkC.T1 with better efficacy than TrkC-FL). MAb **1E11** selectively activates TrkC.T1. MAb **2B7** selectively activates TrkC-FL as a biased agonist, promoting survival but not differentiation (this paper and Guillemard et al., 2010). MAbs **2G5** and **3G5** are non-functional. Small molecules **3Aa** and **1Aa** promote cell survival with low efficacy, and in survival assays they have high efficacy in combination with 0.1 nM NT-3 (^∗^) above each single treatment (this paper and Zaccaro et al., 2005). As single treatment, small molecules **3Aa** and **1Aa** are very efficient at inducing cellular differentiation. **3Aa** does not activate TrkC.T1, but **1Aa** does. Numbers shown are significant (p ≤ 0.05), N.S. means not-significant versus controls; two-tailed t test.

The different ectodomain structures of TrkC-FL and TrkC.T1 made it possible to discover isoform-selective ligands (mAbs, small molecules, and PTP-sigma) that act as agonists, partial agonists, or biased agonists. The ability of these endogenous or artificial ligands to discriminate binding and/or function takes place despite identical primary ectodomain sequence in TrkC and despite the fact that NT-3 does not discriminate between the naturally occurring receptor isoforms.

## Discussion

We report that changes in the intracellular domain primary sequence of a receptor can lead to changes in the ectodomain secondary structure. This finding supports the concept of a bidirectional regulation of receptor conformation: an “outside-to-inside” influence, and an “inside-to-outside” influence. This mechanism increases the known means by which a single gene can generate receptor heterogeneity and signal heterogeneity. We also report on the development of biologically active isoform-selective ligands, even though the primary amino acid sequence of the isoforms is the same and they all bind the natural ligand NT-3.

### Out-To-In versus In-To-Out Control of Receptor Conformation

The outside-to-inside conformational influence has been well studied. Ligand binding at the ectodomain of a receptor can cause conformational changes in the transmembrane or the intracellular domains that are required for protein-protein interaction and activation of signaling (Changeux and Christopoulos, 2016; Dawson et al., 2005; Edelstein and Changeux, 2016; Lemmon et al., 2014). The outside-to-inside effect is promoted by ligand binding, can be orthosteric or allosteric, and may be mirrored by receptor over-expression or by receptor mutations inducing or stabilizing an activated transition state in the receptor.

These structural changes have to permeate the lipid bilayer to be reflected in the intracellular domain. The process is highly regulated by the presence of co-receptors and accessory proteins on the same cell surface (homotypic, acting in *cis*-form) or present in the extracellular matrix or in adjoining cell surfaces (heterotypic, acting in *trans*-form), including matrix proteins, lipids and glycolipids, regulators of membrane fluidity, post-translational modifications, and many other factors (Bublil and Yarden, 2007).

In contrast, the inside-to-outside conformational influence has been less studied. For example, in epidermal growth factor receptors (EGFR) oncogenic mutations or intracellular binding of tyrosine kinase inhibitors can result in altered EGF binding affinity (Macdonald-Obermann and Pike, 2018), potentially through changes in the aggregated state of the receptors or in the ectodomain conformation. Other examples include agents that distinguish between targets that have single amino acid changes, “smart drugs” such as selective c-Src or c-Abl inhibitors (Shawver et al., 2002), or conformationally sensitive mAbs that bind to G-coupled receptors (Heimann et al., 2017) or EGFR family members (Lee et al., 2015), or mAbs and ligands that bind preferentially to homodimers or oligomers (Lemmon and Schlessinger, 2010; Lemmon et al., 2014) or heterodimers (Sweeney et al., 2000). These reports differ from our work because in those cases the receptors have an oncogenic mutation, are pre-activated or over-expressed, or bear the impact of allosteric mechanisms driven by receptor aggregation or by associated co-receptors.

The inside-to-outside effect we report here is independent of ligand binding, independent of changes in primary amino acid sequence or oncogenic transformation, and independent of overexpression or activation. Rather, the effect is caused by alternative splicing of intracellular domains, a process that occurs naturally and is relevant to disease states. Indeed, the alternative splicing of TrkC mRNA impacts in the physiology of many tissues and is implicated in disease onset and disease progression (Bai et al., 2010; Brahimi et al., 2016; Dorsey et al., 2006; Galan et al., 2017; Yanpallewar et al., 2012), highlighting the biological relevance.

### Mechanism of the In-To-Out Control of Ectodomain Conformation

The mechanism of inside-to-outside conformational regulation we report implicates receptor association with PDI in the ER, leading to reorganization of disulfide bridges. The conformations of TrkC-FL (and TrkC-Δ-ICD) differ from that of TrkC.T1. TrkC.T1 has disulfides that are formed differently, as determined in proteolytic mapping studies.

The ER is an oxidizing environment and disulfide bonds initially form spontaneously, but often the first bonds to form are not the correct pair, and spontaneous bonding may not form all of the disulfide bonds required. Breaking and re-forming disulfide bonds in the correct combinations is then one rate-limiting step for folding. PDI accelerates rearrangement of the disulfides so the protein can advance from higher free energy intermediates to the final state, which has the lowest free energy (Braakman and Hebert, 2013; Wallis and Freedman, 2013).

Our data support the notion that the native state of the TrkC-FL isoform (and TrkC-Δ-ICD) is a kinetically trapped folding intermediate that has little contribution by PDI. This conformation is different from the TrkC.T1 native state, an isoform where PDI can break and re-form disulfides more efficiently, to reach the final native state. Hence, the energy barrier between TrkC-FL and TrkC.T1 prevents TrkC-FL from adopting the TrkC.T1 native state.

It is noteworthy that, although the main paradigm studied here is a PDI-mediated impact on the structure of TrkC ectodomains, other differences may arise from differential ER/Golgi transit time, such as differential glycosylation. The potentially confounding interference of other post-translational modifications are, however, excluded in controls in which mild reduction of the TrkC.T1 disulfide bonds generates the TrkC-FL intermediate structure (at least as far as mAb binding is concerned).

Association of PDI with proteins transiting the ER is not unique to TrkC, and the impact of its thiol reductase and isomerase activity have been reported for Integrins, ADAM17 metalloprotease, and thyroid hormone receptors, where PDI association causes changes in their function (Campos et al., 2018; Cho et al., 2012; Lahav et al., 2000). However, to our knowledge a differential impact of PDI on an isoform ectodomain structure has never been reported.

### Selective Interaction of TrkC Isoforms with Endogenous Ligands

Initially, we explored the concept that artificial ligands (mAbs and small molecules) may discriminate between the TrkC-FL and TrkC.T1 isoforms. Having succeeded, we then asked whether natural ligands exist that also do this. Two lines of thought prompted this hypothesis. First, the isoforms are naturally occurring and the mAbs recognize the different isoform conformations *in vivo*. Second, if isoforms do not have functionally selective and biased endogenous natural ligands we would be left with the apparent paradox of simultaneous activation of beneficial and toxic pathways by a single ligand, NT-3.

NT-3 is a native growth factor that binds to TrkC and does not discriminate functionally between the isoforms. Another ligand is PTP-sigma, which associates in a heterotypic fashion (on the membrane of a neighboring cell). PTP-sigma binds to TrkC-FL, TrkC.T1, or TrkC-Δ-ICD and can induce synaptic reorganization by causing clustering any of the TrkC isoforms at the synapse, in an NT-3-independent and in a TrkC intracellular domain-independent manner (Coles et al., 2014; Takahashi et al., 2011).

However, functionally PTP-sigma can activate TrkC-FL to promote cell survival signals but without promoting neuritogenic signals or TrkC.T1-mediated promotion of TNF-α production. This is an example of an endogenous TrkC ligand that is a biased agonist, and selectively activates only one of the activities measured, and only in one isoform.

Considering that several splice and glycosylation isoforms of cell surface PTP-sigma are known (Pulido et al., 1995) there may be a high number of permutations of TrkC-PTP-sigma heterotypic interactions. Moreover, we speculate that there may be other TrkC interactors or accessory proteins, homotypic and heterotypic, that could regulate or discriminate between TrkC isoforms in terms of binding or function. These include, for example, p75^NTR^ (Ivanisevic et al., 2003; Maliartchouk and Saragovi, 1997; Zaccaro et al., 2001), which can regulate synaptogenesis (Hapner et al., 1998), ARMS (Zampieri and Chao, 2006), sortilin (Vaegter et al., 2011), or G-coupled receptors (Lee and Chao, 2001; Zampieri and Chao, 2006).

Conformational heterogeneity in isoforms, and a corresponding heterogeneity in their ligands and their interactions, may be an evolutionary strategy that is able to generate numerous combinations or permutations from a small number of gene products, to regulate a high set of biological processes. Ongoing work beyond the scope of this report is studying the relationship of each TrkC isoform with these other proteins, as well as the relationship of PDI with each of these proteins.

### Exploiting Ectodomain Conformation for Development of Pharmacological Ligands

We show that it is possible to produce mAbs and small molecules that bind selectively to the ectodomain of naturally occurring TrkC receptor isoforms to differentially activate each isoform. This, in spite of the receptors having identical ectodomain primary amino acid sequences.

These results show that it is possible to differentially target receptor isoforms that play different roles in disease states, to specifically modulate their activity. This is relevant from a translational medicine viewpoint. The artificial agonists reported here could provide better alternatives than NT-3 for treatment of pathologies by selectively targeting the TrkC-FL to promote cell survival, while avoiding activation of TrkC.T1, which drives TNF-α expression to neurotoxic levels (Bai et al., 2010; Brahimi et al., 2016; Galan et al., 2017). The anti-TrkC.T1 mAbs may be developed further as antagonists or may be used to eliminate TrkC.T1-expressing cells (which produce TNF-α) at the onset of degenerative diseases such as glaucoma, retinitis pigmentosa, and ALS (Saragovi et al., 2019) (e.g., Müller glial cells in the retina or activated glia in spinal cord).

### Control of Ectodomain Conformation in Receptors

The concept of in-to-out control of receptor conformation may be extended to understanding the many receptors that are alternatively spliced, whether wild-type or oncogenic mutant forms. For example, regarding isoforms in other Trk-family receptors, TrkB has TrkB.T1 and TrkB-T-Shc isoforms generated by use of exon 16 or exon 19, respectively, with alternative polyadenylation signals and translational stops. Deletion of TrkB.T1 is beneficial in a mouse model of ALS (Quarta et al., 2018; Yanpallewar et al., 2012) and in a Down's syndrome mouse model (Dorsey et al., 2006). TrkA has ectodomain isoforms, one of which is relevant to the human disease anhidrosis (Miura et al., 2000), and implicated in responses to noxious stimuli, as well as neuropathic pain (Hefti et al., 2006). Expression of the TrkAIII isoform and other isoforms are correlated with aggressiveness in some type of tumors (Gimm et al., 2001; Tacconelli et al., 2005). All these may be potentially targeted by exploiting conformational differences that may exist in the family.

### On the Physiological Role of Receptor Isoforms and Their Ligands

Initially, we explored the concept that artificial ligands (mAbs and small molecules) may discriminate between the TrkC-FL and TrkC.T1 isoforms. Having succeeded, we then asked whether also isoform selective natural ligands exist, given that the receptors are naturally occurring. Otherwise we would be left with the apparent paradox of simultaneous activation of beneficial and toxic pathways by NT-3. The ligand PTP-sigma was an obvious choice, as its role in TrkC-mediated synaptic reorganization was published to be promoted through either isoform, and it is independent of a TrkC intracellular domain or any known direct signal transduction (Coles et al., 2014; Takahashi et al., 2011) and no isoform differences were reported for that specific endpoint. However, to our surprise no other endpoints had been reported for PTP-sigma acting via TrkC (i.e., no cell survival or neuronal differentiation were reported).

In our studies using additional endpoints we find that PTP-sigma can discriminate functionally between TrkC isoforms, even though it binds to all isoforms. PTP-sigma activates TrkC-FL as a biased agonist, with distinct kinetics that promote cell survival signals but without activating neuritogenic signals. Moreover PTP-sigma does not activate the endpoint of TrkC.T1-mediated promotion of TNF-α to neurotoxic levels (Brahimi et al., 2016; Galan et al., 2017).

Regarding a role for TrkC-FL and TrkC.T1, neuronal development is one case where each isoform and their ligands may play unique roles. This heterogeneity in receptors and ligands may be a mechanism for modulating signals in TrkC populations, acting as a rheostat to bias signals (e.g., to survival or differentiation) or to regulate or stabilize synapses that arise *de novo* during the process of neuronal plasticity.

Although both TrkC-FL and TrkC.T1 receptors are expressed during CNS development (Menn et al., 1998), they differ in spatiotemporal and cellular compartmentalization (Menn et al., 2000). The distinct intracellular signaling pathways (phospholipase C, Ras, PI3 for TrkC-FL; tamalin-Arfs and Rac1 GTPases for TrkC.T1) might explain the different roles in synaptic development and maturation (Rizzo et al., 2018) (Kronschlager et al., 2016) (Oshima et al., 2009).

For example, TNF-α mediates synaptic loss (Henstridge et al., 2019) and in disease (depending on the levels) can contribute to functional changes in synaptic plasticity in a deleterious (Kronschlager et al., 2016; Rizzo et al., 2018) or protective manner (Oshima et al., 2009). Hence, PTP-sigma⋅TrkC.T1 promotion of TNF-α could balance NT-3⋅TrkC-FL signals. Indeed, neurons expressing TrkC-FL showed enhancement of major axonal processes, whereas the truncated isoform reduced elongation of major processes but increased their branching (Ichinose and Snider, 2000). This, in part, may also be dependent on co-expression of p75 co-receptors (Hapner et al., 1998). It is also noteworthy that, unrelated to TrkC activity, PTP-sigma also regulates axonal growth after injury and inhibits axon regeneration (Sapieha et al., 2005; Thompson et al., 2003).

### Conclusions

The development of mAb and small molecule artificial ligands that discriminate TrkC isoforms, as well as the finding of an endogenous ligand PTP-sigma that discriminates TrkC isoforms, enabled binding and functional assays that support the notion of conformational differences at the ectodomain. Conformational differences are supported also by protein mapping and biochemical characterization of the disulfide binding patterns and association with PDI.

Conformational characterization is not feasible using techniques for structural studies (e.g., crystallography or NMR on soluble receptor isoforms) because the absence of intracellular domain yields a single ectodomain conformation, e.g., TrkC-Δ-ICD is identical to TrkC-FL as far as recognition by all the ligands and the interaction with PDI is concerned. In that regard, our results yield a cautionary note with respect to the structure of the ectodomains of cell surface receptors that are engineered at their intracellular domains or that are engineered to be secreted as soluble proteins. The engineering could lead to alternate ectodomain conformation or to disulfide bonding that is entropically driven (Braakman and Hebert, 2013; Kozlov et al., 2010b; Wallis and Freedman, 2013) and may lose any kinetically driven conformations or disulfide bonding. The conformation of such engineered receptors may not correspond to the conformation on the cell membrane, yet the proteins could still be able to bind to ligands and mAbs. CryoEM techniques or the TM cleavage followed by purification of receptor isoforms after cell surface expression may be possible approaches to extend this study.

This paper validates the concept that naturally occurring variants of a receptor intracellular domain can impact on the conformation of the ectodomain (in-to-out effect) to enable isoform-selective ligands with biased bioactivity, both naturally occurring ligands (PTP-sigma) or artificial ligands (mAbs or small molecules). This strategy may be applied to other receptors.

### Limitations of the Study

1 Structural studies using soluble TrkC.T1 isoform ectodomain are not feasible. The absence of an intracellular domain (as we demonstrate for TrkC-Δ-ICD) yields an ectodomain conformation that folds like the TrkC-FL.
2 Purification of the ectodomain of receptor isoforms cleaved from the cell surface may be a suitable approach but was not attempted. Perhaps cryo-EM on membrane proteins might be useful, but the resolution is unlikely to be adequate.
3 The presence of different ectodomain protein structures for TrkC-FL and TrkC.T1 (recognized by the different mAbs) was shown *in vivo* to change in disease, but most of the conclusions regarding signaling pathways are based on the use of cell lines.
4 Biochemical characterization of the isoforms and their association with PDI is based on the use of cell lines.
5 The precise pairs of disulfide-bonded cysteines were not identified in TrkC.T1.
6 Receptor isoform internalization and recycling, and glycosylation, were not evaluated.

### Resource Availability

#### Lead Contact

Further information and request for resources and reagents should be directed to and will be fulfilled by the Lead Contact, Dr. H. Uri Saragovi (uri.saragovi@mcgill.ca).

#### Materials Availability

Reagents generated in this study will be made available on request, but we may require a payment of costs and/or a completed Materials Transfer Agreement.

#### Data and Code Availability

This study did not generate/analyze datasets or code. Original data for figures and tables in the paper are available from the corresponding author on request.

## Methods

All methods can be found in the accompanying Transparent Methods supplemental file.

## Supplemental Information

Document S1. Transparent Methods and Figures S1–S5

## References

[bib1] Bai Y., Shi Z., Zhuo Y., Liu J., Malakhov A., Ko E., Burgess K., Schaefer H., Esteban P.F., Tessarollo L. (2010). In glaucoma the upregulated truncated TrkC.T1 receptor isoform in glia causes increased TNF-alpha production, leading to retinal ganglion cell death. Invest. Ophthalmol. Vis. Sci..

[bib3] Braakman I., Hebert D.N. (2013). Protein folding in the endoplasmic reticulum. Cold Spring Harb Perspect. Biol..

[bib4] Brahimi F., Liu J., Malakhov A., Chowdhury S., Purisima E.O., Ivanisevic L., Caron A., Burgess K., Saragovi H.U. (2010). A monovalent agonist of TrkA tyrosine kinase receptors can be converted into a bivalent antagonist. Biochim. Biophys. Acta.

[bib5] Brahimi F., Maira M., Barcelona P.F., Galan A., Aboulkassim T., Teske K., Rogers M.L., Bertram L., Wang J., Yousefi M. (2016). The paradoxical signals of two TrkC receptor isoforms supports a rationale for novel therapeutic strategies in ALS. PLoS One.

[bib6] Bublil E.M., Yarden Y. (2007). The EGF receptor family: spearheading a merger of signaling and therapeutics. Curr. Opin. Cell Biol..

[bib7] Campos J.L.O., Doratioto T.R., Videira N.B., Ribeiro Filho H.V., Batista F.A.H., Fattori J., Indolfo N.C., Nakahira M., Bajgelman M.C., Cvoro A. (2018). Protein disulfide isomerase modulates the activation of thyroid hormone receptors. Front. Endocrinol. (Lausanne).

[bib8] Changeux J.P., Christopoulos A. (2016). Allosteric modulation as a unifying mechanism for receptor function and regulation. Cell.

[bib9] Chen D., Brahimi F., Angell Y., Li Y.C., Moscowicz J., Saragovi H.U., Burgess K. (2009). Bivalent peptidomimetic ligands of TrkC are biased agonists and selectively induce neuritogenesis or potentiate neurotrophin-3 trophic signals. ACS Chem. Biol..

[bib10] Cho J., Kennedy D.R., Lin L., Huang M., Merrill-Skoloff G., Furie B.C., Furie B. (2012). Protein disulfide isomerase capture during thrombus formation in vivo depends on the presence of beta3 integrins. Blood.

[bib11] Coles C.H., Mitakidis N., Zhang P., Elegheert J., Lu W., Stoker A.W., Nakagawa T., Craig A.M., Jones E.Y., Aricescu A.R. (2014). Structural basis for extracellular cis and trans RPTPsigma signal competition in synaptogenesis. Nat. Commun..

[bib12] Dawson J.P., Berger M.B., Lin C.C., Schlessinger J., Lemmon M.A., Ferguson K.M. (2005). Epidermal growth factor receptor dimerization and activation require ligand-induced conformational changes in the dimer interface. Mol. Cell Biol..

[bib13] Deinhardt K., Chao M.V. (2014). Trk receptors. Handb Exp. Pharmacol..

[bib14] Dorsey S.G., Renn C.L., Carim-Todd L., Barrick C.A., Bambrick L., Krueger B.K., Ward C.W., Tessarollo L. (2006). In vivo restoration of physiological levels of truncated TrkB.T1 receptor rescues neuronal cell death in a trisomic mouse model. Neuron.

[bib15] Edelstein S.J., Changeux J.P. (2016). Biased allostery. Biophys. J..

[bib16] Esteban P.F., Yoon H.Y., Becker J., Dorsey S.G., Caprari P., Palko M.E., Coppola V., Saragovi H.U., Randazzo P.A., Tessarollo L. (2006). A kinase-deficient TrkC receptor isoform activates Arf6-Rac1 signaling through the scaffold protein tamalin. J. Cell Biol..

[bib17] Galan A., Jmaeff S., Barcelona P.F., Brahimi F., Sarunic M.V., Saragovi H.U. (2017). In retinitis pigmentosa TrkC.T1-dependent vectorial Erk activity upregulates glial TNF-alpha, causing selective neuronal death. Cell Death Dis.

[bib18] Geva Y., Schuldiner M. (2014). The back and forth of cargo exit from the endoplasmic reticulum. Curr. Biol..

[bib19] Gimm O., Dziema H., Brown J., de la Puente A., Hoang-Vu C., Dralle H., Plass C., Eng C. (2001). Mutation analysis of NTRK2 and NTRK3, encoding 2 tyrosine kinase receptors, in sporadic human medullary thyroid carcinoma reveals novel sequence variants. Int. J. Cancer.

[bib20] Guillemard V., Ivanisevic L., Garcia A.G., Scholten V., Lazo O.M., Bronfman F.C., Saragovi H.U. (2010). An agonistic mAb directed to the TrkC receptor juxtamembrane region defines a trophic hot spot and interactions with p75 coreceptors. Dev. Neurobiol..

[bib21] Hapner S.J., Boeshore K.L., Large T.H., Lefcort F. (1998). Neural differentiation promoted by truncated trkC receptors in collaboration with p75(NTR). Dev. Biol..

[bib22] Hefti F.F., Rosenthal A., Walicke P.A., Wyatt S., Vergara G., Shelton D.L., Davies A.M. (2006). Novel class of pain drugs based on antagonism of NGF. Trends Pharmacol. Sci..

[bib23] Heimann A.S., Gupta A., Gomes I., Rayees R., Schlessinger A., Ferro E.S., Unterwald E.M., Devi L.A. (2017). Generation of G protein-coupled receptor antibodies differentially sensitive to conformational states. PLoS One.

[bib24] Henstridge C.M., Hyman B.T., Spires-Jones T.L. (2019). Beyond the neuron-cellular interactions early in Alzheimer disease pathogenesis. Nat. Rev. Neurosci..

[bib25] Ichinose T., Snider W.D. (2000). Differential effects of TrkC isoforms on sensory axon outgrowth. J. Neurosci. Res..

[bib26] Ivanisevic L., Banerjee K., Saragovi H.U. (2003). Differential cross-regulation of TrkA and TrkC tyrosine kinase receptors with p75. Oncogene.

[bib27] Kozlov G., Azeroual S., Rosenauer A., Maattanen P., Denisov A.Y., Thomas D.Y., Gehring K. (2010). Structure of the catalytic a(0)a fragment of the protein disulfide isomerase ERp72. J. Mol. Biol..

[bib28] Kozlov G., Maattanen P., Thomas D.Y., Gehring K. (2010). A structural overview of the PDI family of proteins. FEBS J..

[bib29] Kronschlager M.T., Drdla-Schutting R., Gassner M., Honsek S.D., Teuchmann H.L., Sandkuhler J. (2016). Gliogenic LTP spreads widely in nociceptive pathways. Science.

[bib30] Lahav J., Gofer-Dadosh N., Luboshitz J., Hess O., Shaklai M. (2000). Protein disulfide isomerase mediates integrin-dependent adhesion. FEBS Lett..

[bib31] Lee F., Chao M. (2001). Activation of Trk neurotrophin receptors in the absence of neurotrophins. Proc. Natl. Acad. Sci. U S A.

[bib32] Lee R.J., Liu C.W., Harty C., McCracken A.A., Latterich M., Romisch K., DeMartino G.N., Thomas P.J., Brodsky J.L. (2004). Uncoupling retro-translocation and degradation in the ER-associated degradation of a soluble protein. EMBO J..

[bib33] Lee S., Greenlee E.B., Amick J.R., Ligon G.F., Lillquist J.S., Natoli E.J., Hadari Y., Alvarado D., Schlessinger J. (2015). Inhibition of ErbB3 by a monoclonal antibody that locks the extracellular domain in an inactive configuration. Proc. Natl. Acad. Sci. U S A.

[bib34] Lemmon M.A., Schlessinger J. (2010). Cell signaling by receptor tyrosine kinases. Cell.

[bib35] Lemmon M.A., Schlessinger J., Ferguson K.M. (2014). The EGFR family: not so prototypical receptor tyrosine kinases. Cold Spring Harb. Perspect. Biol..

[bib36] Macdonald-Obermann J.L., Pike L.J. (2018). Allosteric regulation of epidermal growth factor (EGF) receptor ligand binding by tyrosine kinase inhibitors. J. Biol. Chem..

[bib37] Maliartchouk S., Saragovi H.U. (1997). Optimal nerve growth factor trophic signals mediated by synergy of TrkA and p75 receptor-specific ligands. J. Neurosci..

[bib38] Matsusaki M., Kanemura S., Kinoshita M., Lee Y.H., Inaba K., Okumura M. (2020). The protein disulfide isomerase family: from proteostasis to pathogenesis. Biochim. Biophys. Acta Gen. Subj..

[bib39] Menn B., Timsit S., Calothy G., Lamballe F. (1998). Differential expression of TrkC catalytic and noncatalytic isoforms suggests that they act independently or in association. J. Comp. Neurol..

[bib40] Menn B., Timsit S., Represa A., Mateos S., Calothy G., Lamballe F. (2000). Spatiotemporal expression of noncatalytic TrkC NC2 isoform during early and late CNS neurogenesis: a comparative study with TrkC catalytic and p75NTR receptors. Eur. J. Neurosci..

[bib41] Michelsen K., Yuan H., Schwappach B. (2005). Hide and run. Arginine-based endoplasmic-reticulum-sorting motifs in the assembly of heteromultimeric membrane proteins. EMBO Rep..

[bib42] Miura Y., Mardy S., Awaya Y., Nihei K., Endo F., Matsuda I., Indo Y. (2000). Mutation and polymorphism analysis of the TRKA (NTRK1) gene encoding a high-affinity receptor for nerve growth factor in congenital insensitivity to pain with anhidrosis (CIPA) families. Hum. Genet..

[bib43] Oshima T., Lee S., Sato A., Oda S., Hirasawa H., Yamashita T. (2009). TNF-alpha contributes to axonal sprouting and functional recovery following traumatic brain injury. Brain Res..

[bib44] Palko M.E., Coppola V., Tessarollo L. (1999). Evidence for a role of truncated trkC receptor isoforms in mouse development. J. Neurosci..

[bib45] Pulido R., Serra-Pages C., Tang M., Streuli M. (1995). The LAR/PTP delta/PTP sigma subfamily of transmembrane protein-tyrosine-phosphatases: multiple human LAR, PTP delta, and PTP sigma isoforms are expressed in a tissue-specific manner and associate with the LAR-interacting protein LIP.1. Proc. Natl. Acad. Sci. U S A.

[bib46] Quarta E., Fulgenzi G., Bravi R., Cohen E.J., Yanpallewar S., Tessarollo L., Minciacchi D. (2018). Deletion of the endogenous TrkB.T1 receptor isoform restores the number of hippocampal CA1 parvalbumin-positive neurons and rescues long-term potentiation in pre-symptomatic mSOD1(G93A) ALS mice. Mol. Cell Neurosci..

[bib47] Rizzo F.R., Musella A., De Vito F., Fresegna D., Bullitta S., Vanni V., Guadalupi L., Stampanoni Bassi M., Buttari F., Mandolesi G. (2018). Tumor necrosis factor and interleukin-1beta modulate synaptic plasticity during neuroinflammation. Neural Plast..

[bib48] Sapieha P.S., Duplan L., Uetani N., Joly S., Tremblay M.L., Kennedy T.E., Di Polo A. (2005). Receptor protein tyrosine phosphatase sigma inhibits axon regrowth in the adult injured CNS. Mol. Cell Neurosci..

[bib49] Saragovi H.U., Galan A., Levin L.A. (2019). Neuroprotection: pro-survival and anti-neurotoxic mechanisms as therapeutic strategies in neurodegeneration. Front. Cell Neurosci..

[bib50] Saragovi H.U., Hamel E., Di Polo A. (2009). A neurotrophic rationale for the therapy of neurodegenerative disorders. Curr. Alzheimer Res..

[bib51] Saragovi H.U., Zheng W., Maliartchouk S., DiGugliemo G.M., Mawal Y.R., Kamen A., Woo S.B., Cuello A.C., Debeir T., Neet K.E. (1998). A TrkA-selective, fast internalizing nerve growth factor-antibody complex induces trophic but not neuritogenic signals. J. Biol. Chem..

[bib53] Segal R.A. (2003). Selectivity in neurotrophin signaling: theme and variations. Annu. Rev. Neurosci..

[bib54] Sendtner M., Holtmann B., Hughes R.A. (1996). The response of motoneurons to neurotrophins. Neurochem. Res..

[bib55] Shawver L.K., Slamon D., Ullrich A. (2002). Smart drugs: tyrosine kinase inhibitors in cancer therapy. Cancer Cell.

[bib56] Sweeney C., Lai C., Riese D.J., Diamonti A.J., Cantley L.C., Carraway K.L. (2000). Ligand discrimination in signaling through an ErbB4 receptor homodimer. J. Biol. Chem..

[bib57] Szobota S., Mathur P.D., Siegel S., Black K., Saragovi H.U., Foster A.C. (2019). BDNF, NT-3 and Trk receptor agonist monoclonal antibodies promote neuron survival, neurite extension, and synapse restoration in rat cochlea ex vivo models relevant for hidden hearing loss. PLoS One.

[bib58] Tacconelli A., Farina A.R., Cappabianca L., Gulino A., Mackay A.R. (2005). Alternative TrkAIII splicing: a potential regulated tumor-promoting switch and therapeutic target in neuroblastoma. Future Oncol..

[bib59] Takahashi H., Arstikaitis P., Prasad T., Bartlett T.E., Wang Y.T., Murphy T.H., Craig A.M. (2011). Postsynaptic TrkC and presynaptic PTPsigma function as a bidirectional excitatory synaptic organizing complex. Neuron.

[bib60] Tessarollo L., Tsoulfas P., Donovan M.J., Palko M.E., Blair-Flynn J., Hempstead B.L., Parada L.F. (1997). Targeted deletion of all isoforms of the trkC gene suggests the use of alternate receptors by its ligand neurotrophin-3 in neuronal development and implicates trkC in normal cardiogenesis. Proc. Natl. Acad. Sci. U S A.

[bib61] Thompson K.M., Uetani N., Manitt C., Elchebly M., Tremblay M.L., Kennedy T.E. (2003). Receptor protein tyrosine phosphatase sigma inhibits axonal regeneration and the rate of axon extension. Mol. Cell Neurosci..

[bib62] Tsoulfas P., Soppet D., Escandon E., Tessarollo L., Mendoza-Ramirez J.L., Rosenthal A., Nikolics K., Parada L.F. (1993). The rat trkC locus encodes multiple neurogenic receptors that exhibit differential response to neurotrophin-3 in PC12 cells. Neuron.

[bib63] Urfer R., Tsoulfas P., O'Connell L., Hongo J.-A., Zhao W., Presta L.G. (1998). High resolution mapping of the binding site of TrkA for nerve growth factor and TrkC for neurotrophin-3 on the second immunoglobulin-like domain of the Trk receptors. J. Biol. Chem..

[bib64] Urfer R., Tsoulfas P., O'Connell L., Shelton D., Parada L., Presta L. (1995). An immunoglobulin-like domain determines the specificity of neurotrophin receptors. EMBO J..

[bib65] Vaegter C.B., Jansen P., Fjorback A.W., Glerup S., Skeldal S., Kjolby M., Richner M., Erdmann B., Nyengaard J.R., Tessarollo L. (2011). Sortilin associates with Trk receptors to enhance anterograde transport and neurotrophin signaling. Nat. Neurosci..

[bib66] Wallis A.K., Freedman R.B. (2013). Assisting oxidative protein folding: how do protein disulphide-isomerases couple conformational and chemical processes in protein folding?. Top. Curr. Chem..

[bib67] Yanpallewar S.U., Barrick C.A., Buckley H., Becker J., Tessarollo L. (2012). Deletion of the BDNF truncated receptor TrkB.T1 delays disease onset in a mouse model of amyotrophic lateral sclerosis. PLoS One.

[bib68] Youn Y.H., Feng J., Tessarollo L., Ito K., Sieber-Blum M. (2003). Neural crest stem cell and cardiac endothelium defects in the TrkC null mouse. Mol. Cell Neurosci..

[bib69] Zaccaro M.C., Ivanisevic L., Perez P., Meakin S.O., Saragovi H.U. (2001). p75 Co-receptors regulate ligand-dependent and ligand-independent Trk receptor activation, in part by altering Trk docking subdomains. J. Biol. Chem..

[bib70] Zaccaro M.C., Lee H.B., Pattarawarapan M., Xia Z., Caron A., L'Heureux P.J., Bengio Y., Burgess K., Saragovi H.U. (2005). Selective small molecule peptidomimetic ligands of TrkC and TrkA receptors afford discrete or complete neurotrophic activities. Chem. Biol..

[bib71] Zampieri N., Chao M.V. (2006). Mechanisms of neurotrophin receptor signalling. Biochem. Soc. Trans..

[bib72] Zhang Y., Moheban D.B., Conway B.R., Bhattacharyya A., Segal R.A. (2000). Cell surface Trk receptors mediate NGF-induced survival while internalized receptors regulate NGF-induced differentiation. J. Neurosci..

