# Supplementary material for: Alternative Splicing of a Receptor Intracellular Domain Yields Different Ectodomain Conformations, Enabling Isoform-Selective Functional Ligands
Source: iScience. 2020 Aug 10;23(9):101447. doi: 10.1016/j.isci.2020.101447 (PMC7452315; doi:10.1016/j.isci.2020.101447)
Supplement: Document S1. Transparent Methods and Figures S1–S5 [file mmc1.pdf]

iScience, Volume *23*

Supplemental Information

Alternative Splicing of a Receptor Intracellular Domain Yields Different Ectodomain Conformations, Enabling Isoform-Selective Functional Ligands

Fouad Brahimi, Alba Galan, Sean Jmaeff, Pablo F. Barcelona, Nicolas De Jay, Kurt Dejgaard, Jason C. Young, Claudia L. Kleinman, David Y. Thomas, and H. Uri Saragovi

SUPPLEMENTAL FIGURES

Figure S1. 1E11 and 1B2 mAbs bind selectively to upregulated TrkC.T1 protein in sections of ALS spinal cord. Related to Figure 1 and Tables 1 and 2. Spinal cord sections of 12 µm were prepared from G93A ALS mice or wild type mice (ventral horn, lumbar region L2-L3). TrkC.T1 protein (red) was identified using selective anti-TrkC.T1 mAb **1E11** (ectodomain) or anti-TrkC.T1 mAb mAb **1B2** (intracellular domain). Glia is localized using the GFAP marker (green). Pictures were taken using a Leica spinning disc confocal microscope with 63x magnification. **A. 1E11** staining. **B. 1B2** staining. **C.** Negative control in the absence of primary antibody. The insets show a higher magnification of the small white rectangles. Scale bar 7 µm. Similar staining data was obtained from thoracic sections (data not shown).

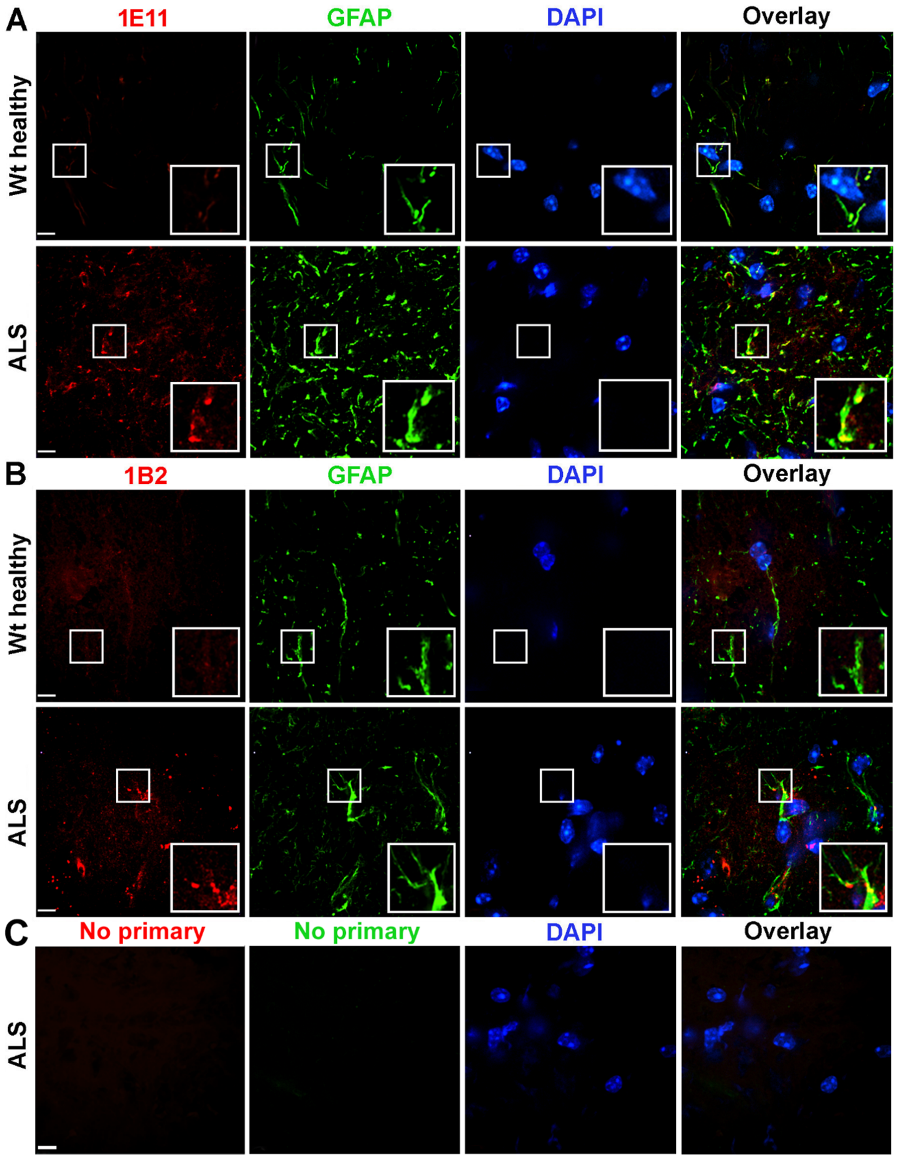

Figure S2. ER retention or export sequences in the intracellular domain of human TrkC isoforms. Related to Figure 2. Underlining indicates the transmembrane domain. Bold indicates the unique spliced-in sequence added to TrkC.T1. Light shading indicates ER exit sequences (7 in TrkC-FL and 4 in TrkC.T1). Dark shading indicates ER retrieval sequences (2 sites in TrkC-FL). TrkC-Δ-ICD does not have an intracellular domain, and has no ER exit or ER retrieval sequences.

hTrkC.T1

FGVSIAVGLAAFACVLLVVLFVMINKYGRRSKFGM
KGPVAVISGEEDSASPLHHINHGITTPSSLDAGPDTV
VIGMTRIPVIENPQYFRQGHNCHKPDT**WVFSNIDN**
HGILNLKDNRDHLVPSTHYIYEEPEVQSGEVSYP
RSHGFREIMLNPISLPGHSKPLNHGIYVEDVNVY
FSKGRHGF

hTrkC-FL

FGVSIAVGLAAFACVLLVVLFVMINKYGRRSKFGM
KGPVAVISGEEDSASPLHHINHGITTPSSLDAGPDTV
VIGMTRIPVIENPQYFRQGHNCHKPDTYVQHIKRRD
IVLKRELGEGAFGKVFLAECYNLSPTKDKMLVAVK
ALKDPTLAARKDFQREAELLTNLQHEHIVKFYGVC
GDGDPLIMVFEYMKHGDLNKFLRAHGPDAMILVD
GQPRQAKGELGLSQMLHIASQIASGMVYLASQHFV
HRDLATRNCLVGANLLVKIGDFGMSRDVYSTDYY
RLFNPSGNDFCIWCEVGGHTMLPIRWMPPESIMYR
KFTTESDVWSFGVILWEIFTYGKQPWFQLSNTEVIE
CITQGRVLERPRVCPKEVYDVMLGCWQREPQQRL
NIKEIYKILHALGKATPIYLDILG

Figure S3. MAb 1E11–promoted increase in TNFα mRNA requires TrkC.T1 expression

Related to Figure 4 and Figure 7B. rMC-1 glial cells expressing TrkC.T1 were infected with scrambled control (white bars) or with pLKO-1 virus (back bars) to silence TrkC.T1 expression (Brahimi et al., 2016). After infection the cells were treated with vehicle (untreated control), mAb 1E11, or NT-3 and LPS controls. TNFα mRNA levels were quantified 6 hours after ligand treatment, by quantitative real-time PCR. Compared to untreated control, all the treatments significantly ($p<0.001$) induced TNFα mRNA in the scrambled virus infected control groups (white bars). Data are mean ± standard deviation. Symbols indicate significance. Silencing TrkC.T1 reduced the effect of mAb 1E11 or NT-3, indicating that these agonists act in a TrkC.T1–dependent manner. Silencing of TrkC.T1 did not affect LPS–induced TNFα mRNA, as expected because LPS acts via Toll-like receptors. The brackets indicate significant differences in ligand-promoted induction of TNFα mRNA (* $p<0.05$. ** $p<0.01$; one-way ANOVA with Tukey-Kramer Multiple comparisons).

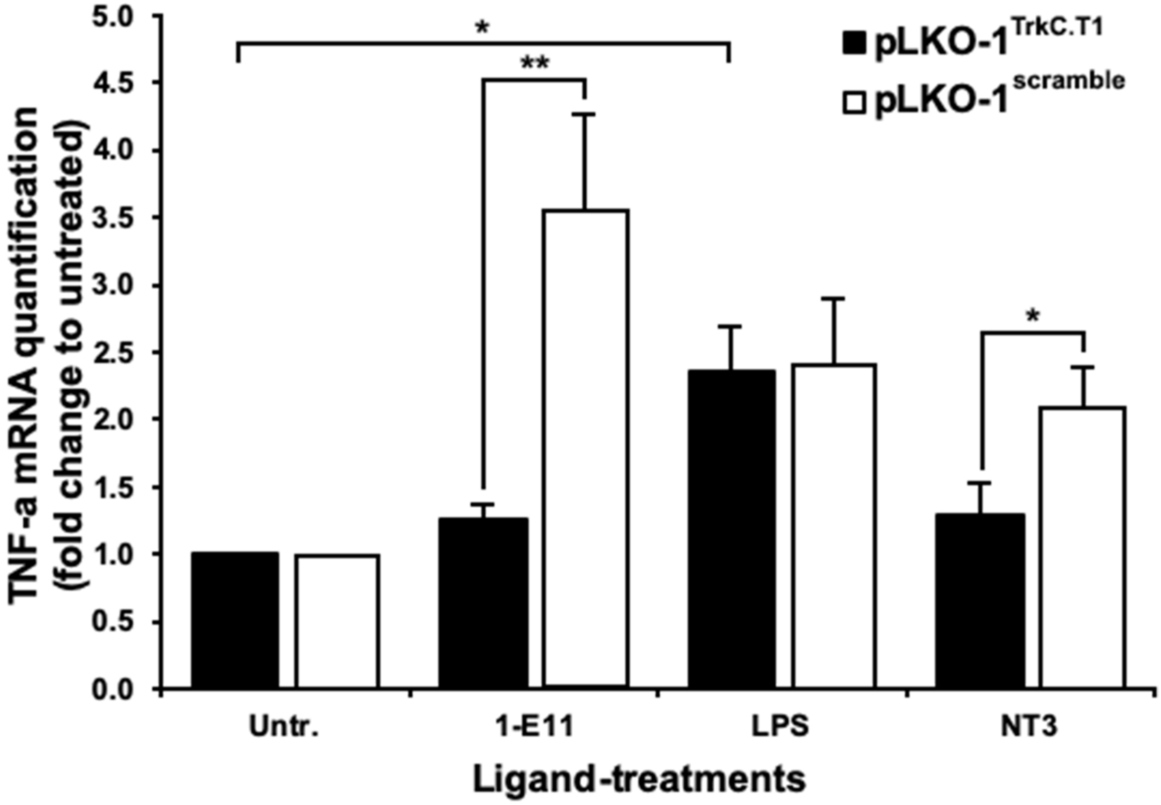

Figure S4. PTP-sigma ectodomain binds to the ectodomain of all TrkC isoforms.

Related to Figures 6 and 7 and Tables 1 and 2. Flow cytometry binding assays using HEK293 cells each stably expressing each isoform. TrkC-FL (black), TrkC.T1 (dashed), or TrkC-Δ-ICD (gray) expressing cells were immunolabeled. **A.** PTP-sigma ectodomain (10 nM). **B.** MAb 2F5 (binding to all isoforms) is a positive control. Irrelevant IgG (red histogram) is a negative control. The same background is seen for all cell types but for simplicity only one background histogram is depicted.

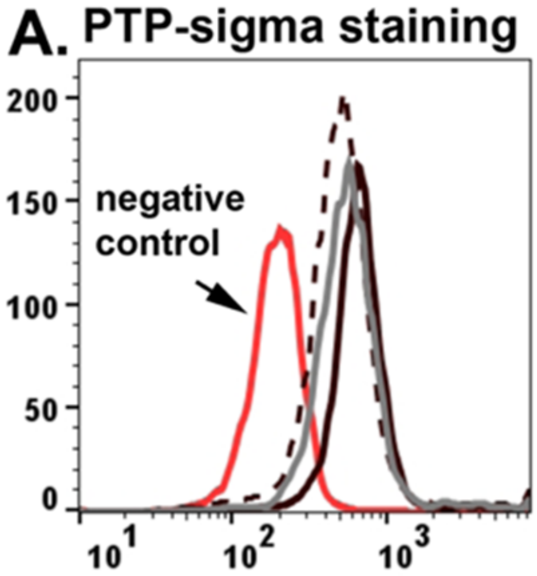

Figure S5. Dose-dependent profiles of TrkC agonists

Related to Figures 4, 5, and 6 and Tables 1 and 2. Dose-response curves of bioactivity were evaluated for all ligands. Shown are the bioactive agents, some inactive ligands are omitted. **A.** TrkC-FL agonist promotion of HEK293-TrkC-FL cell survival in serum-free culture conditions. Potency is determined from the effective concentrations (EC). Mab 2B7 and PTP-sigma have a maximal efficacy that is comparable to optimal NT-3, but they have lower potency (EC_{50} 2 nM for 2B7 and 8 nM for PTP-sigma, *versus* EC_{50} 0.2 nM for NT-3). Mab **2F5** has significantly lower efficacy and potency. Alone, small molecules **3Aa** and **1Aa** have low potency and low efficacy, but respectively enhance the efficacy of 0.1 nM NT-3 from 24% to 65% and 71%, significant versus controls; two-tailed t-test. **B.** TrkC.T1 agonist induction of TNFα in rMC-1-TrkC.T1 cells. Shown are the bioactive agents, some of the inactive ligands are omitted. Data are mean ± standard deviation. Symbols indicate significance * $p \leq 0.05$; two-tailed t-test.

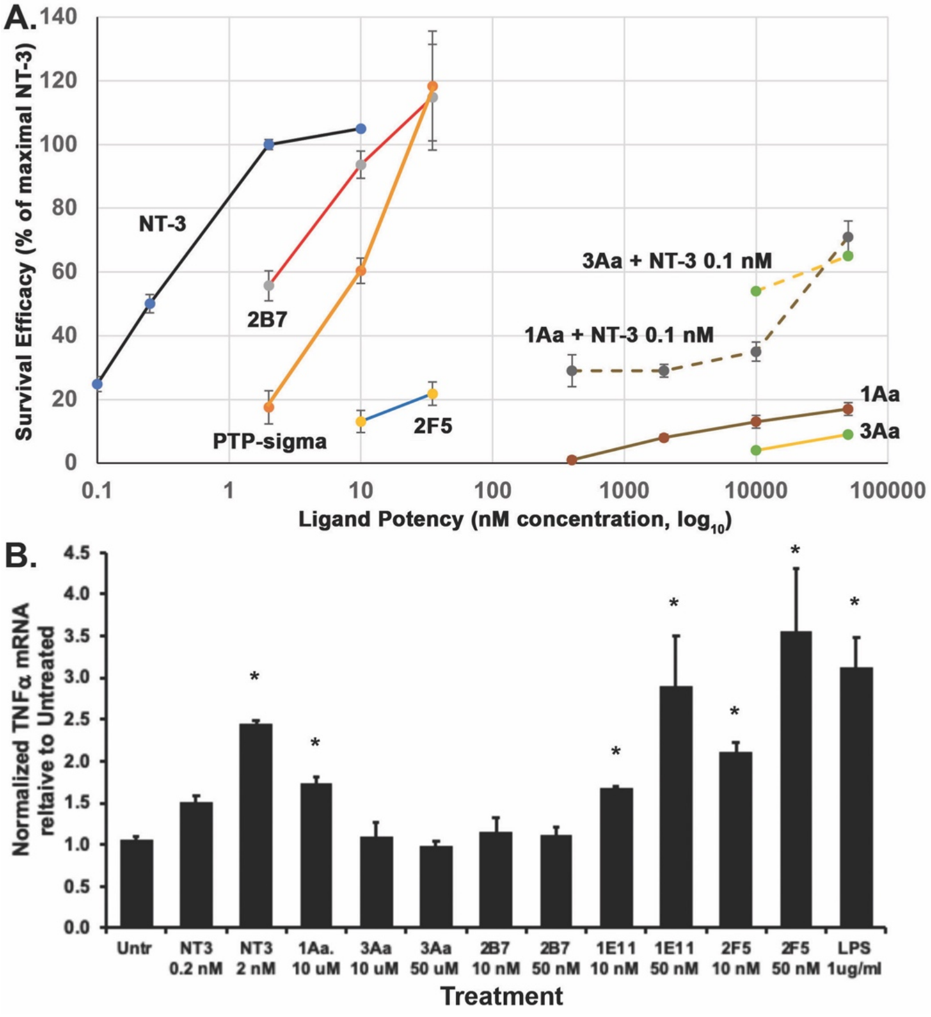

Transparent Methods

Cell lines

Human embryonic kidney HEK 293 (female origin, ATCC) and mouse embryonic fibroblast NIH3T3 cells (ATCC) were transfected with plasmids encoding human full-length *TrkC* (293-TrkC-FL, NIH-TrkC-FL) or with human or rat *TrkC.T1* (293-rTrkC.T1, 293-hTrkC.T1, NIH-TrkC-T1) or with human TrkC.T1$^{\Delta insert}$ construct that was generated by transfection with a pcDNA6 blasticidin-resistant plasmid containing a PCR-generated amplicon encompassing amino acids 1 to 528 of human TrkC.T1 (293-hTrkC-Δ-ICD). The rat glial cell line, rMC-1 has been previously characterized (Sarthy et al, 1998) and expresses low levels of endogenous TrkC.T1, and was also transfected with human TrkC.T1 cDNA (rMC-1-TrkC.T1). Nnr5 cells are a variant of PC12 cells that lost expression of TrkA (the NGF receptor) and were stably transfected with TrkC-FL cDNA and express functional protein on the surface. Stably transfected cell lines were subcloned under drug selection (depending on the vector, 0.5 mg/ml G418, or 2 μg/ml puromycin, or 10 μg/ml blasticidin) and selected based on relatively high expression of receptors (100,000/cell, similar across all lines). Equal expression was verified by flow cytometry, by PCR, and by western blot using our own mAbs or commercial mAb C44H5 antibody that recognizes both isoforms (Cell signalling) (e.g. see (Brahimi et al, 2016)**.** Expression of the receptor levels indicated do not alter cell physiology since there is no significant ligand-independent activation (e.g. see **Figure 6D**, untreated cells), and in serum free conditions all cells die at the same rate as non-transfected cells. All cell lines were mycoplasma free and were regularly tested by using a PCR mycoplasma detection kit (Zentech).

Mice

The animal protocol used was reviewed and approved by the Lady Davis Institute Animal Care Committee and animal experiments were performed according to the guidelines of the Canadian Council on Animal Care. Healthy wild-type female Balb/c mice (10-12 weeks of age, 19-20g) were purchased from Charles River Laboratories (Montreal, Quebec, Canada) and used for the generation of monoclonal antibodies (mAbs). A maximum of five mice per cage were kept in a 12 hr dark-light cycle with food and water *ad libitum*.

Immunization and generation of mAbs

We generated mAbs that bind to TrkC-FL and/or TrkC.T1. One strategy was immunization of mice that do not express TrkC.T1, due to an engineered point mutation at the alternative splicing

site that generates TrkC.T1 mRNA from TrkC-FL mRNA (thereafter named “TrkC.T1 knockout”). The TrkC.T1 knockout mice do not produce TrkC.T1 mRNA or protein (Bai et al., 2010a). Wild type mice were also successfully immunized with live NIH3T3 mouse fibroblasts stably transfected with human TrkC.T1 cDNA (NIH-TrkC.T1), expressing high levels of protein on the cell surface (Brahimi et al, 2016). Other mice were immunized with peptides of the juxtamembrane region of TrkC (present in both TrkC-FL and TrkC.T1 (ESTDNFILFDEVSPTPPI) or the unique neoepitope of TrkC.T1 intracellular domain and only present in TrkC.T1 (GIYVEDVNVYFSKGRHG). All peptides **were** synthesized *in house* and conjugated to KLH as a carrier. Hybridomas were generated from serum–positive mice by fusion of B-cells with SP20 myeloma cells, using standard techniques. Hybridomas were screened in binding assays by IHC and by flow cytometry using cells stably transfected to express either TrkC-FL or TrkC.T1 (e.g. NIH-TrkC.T1 or NIH-TrkC-FL; 293-TrkC.T1 or 293-TrkC-FL), compared to controls wild type non-transfected cells and to cells transfected to express related receptors such as TrkA**.** The mAb 2B7 binds to the TrkC IgG-C2 (D5) domain (Guillemard et al., 2010)**.** All mAbs were purified in protein G columns for use in binding and biological assays.

Biotinylation of NT-3.

NT-3 (Alomone Labs ProSpec) was biotinylated by incubating 2 μM of NT-3 with 400 μM of Sulfo-NHS-LC-Biotin (Thermofisher scientific, cat # 21335) for 40 min at room temperature in PBS (20-fold molar excess of biotin reagent). Biotinylated NT-3 was concentrated, and unbound biotin removed, using a desalting Amicon Ultra-0.5 ml centrifugal filter (Ultracell:10K, Millipore).

Small molecules.

The synthesis and the bioactivity of small molecules **3Aa** and **1Aa** and their fluorescein-conjugated analogs (3Aa-FITC and 1Aa-FITC) have been described (Zaccaro et al, 2005). These agents are reported agonists of TrkC-FL and promote cell survival and cell differentiation.

Flow cytometry.

The ligands were tested by quantitative flow cytometry (Guillemard et al., 2010). Briefly, 293 or NIH-WT or expressing TrkC-FL or TrkC.T1 cells were resuspended in 0.1 mL of flow cytometry buffer and incubated with the indicated compounds for 30 min at 4°C, and washed three times to remove excess. For mAbs, secondary reagent was anti-mouse IgG conjugated to fluorescein. Cells were then suspended in buffer and analyzed by flow cytometry using Flowjo program. As negative controls, no primary (background fluorescence) or irrelevant mouse IgG were used. Reported are

the mean channel fluorescence (MCF) values of at least two independent experiments. For mild reduction tests, live cells were exposed to DTT (10 mM) for 10 min at room temperature. After washing once, iodoacetamide (15 mM) was added to prevent reformation of disulfide bonds, followed by a second wash. MAb 2B7 binding was then studied by flow cytometry, as described above.

Binding competition assays

Binding competition assays were performed (Guillemard et al., 2010;Barcelona et al, 2015) with agents (mAbs, small molecules, or PTP-sigma) tested as competing the binding of NT-3–biotin, or with NT-3 tested as competing the binding of mAbs, small molecules, or PTP-sigma. Cells expressing the indicated TrkC receptor were pre-incubated with competitor (50 nM to 5 μM) for 15 min in PBS/0.2% BSA/0.1% Na Azide pH 7.3 (FACS buffer) at 4°C. Then, labeled binding agents were added (10 nM) for another 15 min. After a FACS buffer washing steps, cells were incubated with fluorescein-conjugated-secondary agent for 15 min at 4°C (for NT-3–biotin avidin-fluorescein (Sigma), and for the MAbs or PTP-sigma anti-Ig-fluorescein (Sigma)). No secondary was added for the small molecules, as they are directly labeled with a fluorescein molecule. After two washing steps, cells were analyzed immediately by flow cytometry. Mean channel fluorescence (MCF) values of bell-shaped histograms were standardized, with no competition = 100%, and secondary without primary = 0%. % of MCF values are the mean of three independent experiments ± SD.

Fluorescence Microscopy.

Cells expressing TrkC-FL or TrkC.T1 were plated on coverslip. After 24 h, cells were exposed to 3Aa-FITC or 1Aa-FITC (10 μM) for 30 min in flow cytometry buffer on ice. After a washing step, cells attached on coverslip were fixed, washed and mounted. Images were taken with a Leica DM LB 2 microscope equipped with the LAS acquisition software and a Leica DFC350 camera for detection, applying a 63x objective. Images were saved directly in TIF format and adjusted for unbiased brightness and contrast using Adobe Photoshop CS 8.0.

Immunohistochemistry.

Cryosections of WT mice or G93A ALS mice were immunostained with 1E11 mAb or 1B2 mAb and GFAP antibody (Cell Signaling, cat # 3670). Images were taken with a Leica DMI6000 B microscope equipped with the Quorum technologies WaveFX spinning disk confocal microscopy system, the Volocity software and a high dynamic ImagEM EM-CCD camera for detection.

Pictures were acquired as Z-stacks of confocal optical sections applying a 63x objective. Images were exported directly in TIF format and adjusted for unbiased brightness and contrast using Photoshop CS 8.0.

Cell Metabolism/Survival functional assays.

The growth/survival profile of cells were quantified in 96-well plates using the tetrazolium salt reagent 4-[4,5-dimethylthiazol-2-yl]-2,5-diphenyltetrazolium bromide (MTT; Sigma) 48-72 hr after plating; by reading the optical density (OD) (Maliartchouk et al., 1997). NIH-TrkC-FL or 293-TrkC-FL cells were treated with different concentrations of the test agents, or with vehicle, or with NT-3 (positive control). Cells expressing a related receptor TrkA (and its ligand NGF) were used as cellular specificity control. Each individual assay was done in quadruplicate and was repeated n=3 independent times. MTT data were standardized to optimal dose of neurotrophin = 100% survival, and serum-free medium (SFM) = 0% survival, using the formula $[(OD_{test} - OD_{SFM}) \times 100 / (OD_{optimal\ NTF} - OD_{SFM})]$. Only optimal doses are shown, for simplicity, which correspond to 2 nM NT-3, 40 nM for mAbs, 10 μM for small molecules, 35 nM for PTP-sigma.

Immunoprecipitation and Western Blot Analysis

Detergent lysates of cells expressing either TrkC-FL, TrkC-Δ-ICD or TrkC.T1 were immunoprecipitated with anti-pan-TrkC (C44H5, Cell Signaling, cat # 3376). Proteins were eluted from the protein G-agarose beads and resolved by SDS-PAGE in reducing (DTT) or non-reduced conditions. Membranes were blotted with anti-pan-TrkC or with two different anti-PDI mAbs (Cell Signaling, cat# 2446 and Genetex, RL90, cat# GTX22792). Mild reduction assays were performed in NP-40 detergent lysates prepared from 293-TrkC-FL or 293-TrkC.T1 cells. Cleared whole cell lysates were resuspended in SDS-PAGE Laemmli buffer lacking reducing agents. After 1 min of exposure to 90°C, DTT (10 mM) was added to the samples and incubated for 15 min at room temperature. All samples were resolved immediately by SDS-PAGE. After western transfer, membranes were immunoblotted with mAb 2B7 (TrkC-FL specific). Densitometric quantification was done using ImageJ software. * $p<0.05$, ** $p< 0.01$ versus control (n=3 independent experiments).

Biochemical signals

Cell signals were reduced to baseline by culture in minimal media for 2 hours. Then, optimal concentrations of NT-3 (2 nM) or PTP-sigma (35 nM) or control vehicle PBS were added and cells were collected after 0, 5, 12, 30 min of stimulation. Whole cell detergent lysates were prepared

and resolved by SDS-PAGE. After western blotting, membranes were studied with specific mAbs directed to the phosphorylated mediators pAkt (Cell Signallng, # 9271), pTrkC (Millipore, # 05-321), pErk1,2 (Cell Signaling, #4370); or against actin as standard control for loading (Sigma, #A2066). N = 3 assays with independently processed lysates. For simplicity, only relevant ligand concentrations and time-points are shown.

Differentiation Assays

Nnr5 cells are a variant of PC12 cells that lost TrkA expression but retain p75^{NTR} expression. Nnr5-TrkC-FL cells are stably transfected with TrkC-FL cDNA and express protein on the surface. The indicated treatments were added for 72 h and differentiation of adherent cells was scored morphologically as % of cells with neurites (> 2 cell body long). Quantification was done by image analysis, from at least two independent assays.

Upregulation of TNFα by activated TrkC.T1, in quantitative real-time PCR assays

Rat glial rMC-1 cells expressing TrkC.T1 were treated with the indicated agents for 6 hr: **3Aa** or **1Aa** (10 μM), LPS (1 μg/ml) (Shariff et al), NT-3 (2 nM) or mAbs (40 nM), human recombinant PTP-sigma ectodomain (10-50 nM) (R&D Systems 3430-PR-050). The mRNA was prepared from the cells using the qScript RNA cDNA synthesis kit (Quanta). Samples were used for real-time quantitative PCR with primers for rat TNFα and rat RNAs18 (Barcelona and Saragovi, 2015;Brahimi et al., 2016). Data are expressed as the mean ± SEM relative to the untreated group (3 independent experiments, each in triplicate).

Mass spectrometry.

TrkC immunoprecipitates were digested with 6 μl of proteomics-grade Trypsin (Promega) at a concentration of 12 ng/ μl in 50 mM ammonium bicarbonate, overnight, at 37 ^{o}C. Digested peptides were washed off the beads with 200 μl of water, the peptide digests were split into two 100 μl aliquots and transferred to clean Eppendorf tubes. One of the Eppendorf tubes was supplemented with DTT (5 mM) and both tubes were heated to 55° C for half an hour. Subsequently, both tubes were supplemented with iodoacetamide (IAA, 10 mM) and after an hour samples were dried in a speed vacuum. The dried peptides were reconstituted in 25 μl of water supplemented with 0.1% formic acid (FA) and transferred to a 200 μl sample vial. The peptide samples were subjected to LC reverse phase nanoflow chromatography using a Proxeon Easy nLC (Thermo Scientific). The peptides were trapped onto a 2 cm C18 column (Acclaim PepMap 100, Thermo Scientific) and were separated at a flow rate of 350 nl/minute on a 15 cm C18 analytical

nanocolumn (Acclaim PepMap RSLC, Thermo Scientific) with a water/Acetonitrile gradient covering 3%-35% Acetonitrile over 100 min. The eluting peptides were analyzed by an Orbitrap Q-Exactive HF (Thermo Scientific) operating with a duty cycle of 25 MSMS fragment spectra per precursor scan. The resolution was set at 120,000 (scan speed 2 spectra per second) for precursor scans, over mass range of 375-1400 m/z, and 30,000 (scan speed 25 spectra per second) for fragment spectra. The mass spectrometer was operated with a dynamic exclusion set at 6 seconds and maximum trap fill. The acquired spectra were converted into Mascot Generic Files (mgf) using Mascot Distiller (Matrix Sciences) and searched against the Human proteome (Uniprot). Searches were performed using the Mascot proteomics search engine (Matrix Sciences), setting mass tolerance of 6 ppm for precursor ions and 50 mDa for MSMS ions and selecting Carbamidomethyl modification modifications of cysteines as a fixed modification and oxidized Methionine as a variable modification. The Mascot data output was transferred to Scaffold (Proteome Software) for data validation for peptides/proteins with a false discovery rate (FDR) of better than 5%.

Mass spectrometry data analysis

Scaffold v4.8.9 was used to extract the data set of trypsin-generated peptides detected by mass spectrometry in reducing and non-reducing conditions, as well as the coverage of each isoform of TrkC (TrkC.T1, TrkC-FL and TrkC-Δ-ICD). To gain further insights into the covalent structure of the ectodomain of each TrkC isoform, unique pairs of peptides were searched in the reduced conditions whose combined mass (AMU) matches that of peptides detected the non-reduced condition, with an error of up to 10 units. Peptides in reducing conditions were restricted to those whose sequences mapped to the extracellular domain of TrkC (residues 32-429 of NTRK3_HUMAN based on UniProtKB).

Quantification and statistical analysis

The dispersion and precision measures (mean ± SD), the statistical significance and the exact value of “n” are reported in the Figures and Figure Legends. Differences between treated and untreated groups were assessed by two-tailed student t-test. For multiple groups, one-way ANOVA with Tukey-Kramer Multiple Comparisons Test compared different groups. Significance $p<0.05$ (*), $p< 0.01$ (**) and $p< 0.001$ (***).

References for Supplemental and Methods

Barcelona, P.F., and Saragovi, H.U. (2015). A Pro-Nerve Growth Factor (proNGF) and NGF Binding Protein, alpha2-Macroglobulin, Differentially Regulates p75 and TrkA Receptors and Is Relevant to Neurodegeneration Ex Vivo and In Vivo. Mol Cell Biol *35*, 3396-3408.

Brahimi, F., Maira, M., Barcelona, P.F., Galan, A., Aboulkassim, T., Teske, K., Rogers, M.L., Bertram, L., Wang, J., Yousefi, M., *et al.* (2016). The Paradoxical Signals of Two TrkC Receptor Isoforms Supports a Rationale for Novel Therapeutic Strategies in ALS. PLoS One *11*, e0162307.

Guillemard, V., Ivanisevic, L., Garcia, A.G., Scholten, V., Lazo, O.M., Bronfman, F.C., and Saragovi, H.U. (2010). An agonistic mAb directed to the TrkC receptor juxtamembrane region defines a trophic hot spot and interactions with p75 coreceptors. Dev Neurobiol *70*, 150-164.

Maliartchouk, S., and Saragovi, H.U. (1997). Optimal nerve growth factor trophic signals mediated by synergy of TrkA and p75 receptor-specific ligands. J Neurosci *17*, 6031-6037.

Sarthy, V.P., Brodjian, S.J., Dutt, K., Kennedy, B.N., French, R.P., and Crabb, J.W. (1998). Establishment and characterization of a retinal Muller cell line. Invest Ophthalmol Vis Sci *39*, 212-216.

Zaccaro, M.C., Lee, H.B., Pattarawarapan, M., Xia, Z., Caron, A., L'Heureux, P.J., Bengio, Y., Burgess, K., and Saragovi, H.U. (2005). Selective small molecule peptidomimetic ligands of TrkC and TrkA receptors afford discrete or complete neurotrophic activities. Chem Biol *12*, 1015-1028.
